# From Nature to Function: Green Composites Using Camphoric Acid-Based Unsaturated Polyester Resin and Bamboo/Flax Non-Woven Reinforcements

**DOI:** 10.3390/polym17223038

**Published:** 2025-11-17

**Authors:** Slavko Mijatov, Sanja Savić, Saša Brzić, Stefan Ivanović, Milena Simić, Milena Milošević, Aleksandar Marinković

**Affiliations:** 1 Department of Organic Chemical Technology, Faculty of Technology and Metallurgy, University of Belgrade, 11120 Belgrade, Serbia; sseslija@tmf.bg.ac.rs; 2 Department of Materials and Protection, Military Technical Institute, 11030 Belgrade, Serbia; sasabrzic@gmail.com; 3 Department of Chemistry, Institute of Chemistry, Technology and Metallurgy—National Institute of the Republic of Serbia, University of Belgrade, 11000 Belgrade, Serbia; stefan.ivanovic@ihtm.bg.ac.rs; 4 Department of Organic Chemistry, Faculty of Pharmacy, University of Belgrade, 11000 Belgrade, Serbia; milena.simic@pharmacy.bg.ac.rs; 5 Department of Ecology and TechnoEconomics, Institute of Chemistry, Technology and Metallurgy—National Institute of the Republic of Serbia, University of Belgrade, 11000 Belgrade, Serbia; milena.milosevic@ihtm.bg.ac.rs; 6 Department of Organic Chemistry, Faculty of Technology and Metallurgy, University of Belgrade, 11120 Belgrade, Serbia; marinko@tmf.bg.ac.rs

**Keywords:** bio-resin, bio-fibers, non-woven, bio-composites, thermo-mechanical properties

## Abstract

Unsaturated polyester resins (UPRs) were synthesized from camphoric acid and diluted with styrene, partially replaced (up to 30%) by trimethylolpropane triacrylate (TMPTA). Rheological tests showed increased but sustainable viscosity due to TMPTA’s higher polarity. These UPRs served as matrices for composites reinforced with non-woven bamboo and flax mats from recycled waste. Mechanical testing revealed that C_f_-UPR/TMPTA30 exhibited the highest tensile strength (25.2 MPa) and modulus (0.96 GPa), compared to 18.7 MPa and 0.74 GPa for the styrene-based resin, respectively, attributed to greater cross-link density. Bamboo composites showed lower tensile properties (13.6 MPa) due to random fiber orientation and porosity, while flax-reinforced systems, especially C_f_-UPR/TMPTA30–FLAX, reached 42.7 MPa tensile and 95.5 MPa flexural strength, indicating synergy between TMPTA-modified resin and flax fibers. Dynamic-mechanical analysis confirmed stable thermo-mechanical behavior, and water uptake tests showed reduced absorption (by ~10%), suggesting improved fiber/matrix adhesion. SEM images revealed brittle fracture and fiber pull-out in styrene systems, but fiber breakage and ductile textures in TMPTA-based ones, proving better stress transfer. Thermal analysis indicated slightly earlier degradation onset for TMPTA-modified resins but higher char yield in fiber composites. Overall, TMPTA substitution and flax reinforcement enhance the mechanical, interfacial, and thermal properties of bio-based UPRs, supporting sustainable high-performance composites.

## 1. Introduction

The increasing environmental impact of petroleum-based polymers has intensified research into sustainable alternatives, particularly bio-based polyester resins derived from renewable resources. Further it concerns and stringent regulations on synthetic petroleum-based polymers have driven significant research into developing sustainable alternatives [1,2]. Among these, bio-based unsaturated polyester resins have emerged as promising candidates due to their potential to reduce carbon footprint while maintaining comparable mechanical and thermal properties to conventional resins. These resins offer a viable solution to reduce dependency on fossil fuels while maintaining performance in composite applications. Recent studies have demonstrated progress in synthesizing polyester resins from renewable resources, including plant oils (e.g., soybean, castor, and linseed oil), lignin, and glycerol [3,4,5,6]. According to the recent investigations [7,8], trimethylolpropane triacrylate (TMPTA) has been identified as a promising multifunctional acrylate diluent for a partial substitution of styrene (STY), a commercially applied reactive but volatile and hazardous aromatic diluent in unsaturated polyester resin systems. Ongoing efforts to replace styrene in industry are driven by health and environmental concerns, as it is classified by the European Chemical Agency as suspected to be toxic to reproduction, by the US Department of Health and Human Services as reasonably anticipated to be a human carcinogen, and by the US Environmental Protection Agency as a hazardous air pollutant [7,9]. TMPTA incorporation, even at low concentrations, improved cross-link density and mechanical performance—most notably enhancing impact strength and tensile properties—while maintaining comparable thermal properties to conventional styrene-based systems. Rubeš et al. [7] used itaconic acid based UPR system modified with 2 wt.% TMPTA and reported a high ultimate tensile strength of 70.3 ± 1.7 MPa, alongside a significantly tighter network (higher crosslink density), as revealed by dynamic-mechanical analysis (DMA)—clearly demonstrating that even low TMPTA additions can enhance both mechanical integrity and network structure compared to formulations using only methyl methacrylate (MMA) or no secondary cross-linker. Grimalt et al. [8] evaluated TMPTA alongside other reactive diluents (e.g., MMA and limonene) and found that even low loadings of TMPTA (e.g., ~5 wt.%) contributed to favorable thermo-mechanical behavior, suggesting it as a promising renewable alternative to STY in UPR formulations, but also led to an increase in the resin’s viscosity. However, according to our best knowledge, no study related to the use of a higher mass fraction of TMPTA in polyester system has been found.

The natural fibers offer several advantages compared to synthetic ones, such as low energy consumption for the manufacture of natural non-woven fibers, non-abrasive behavior, unlimited availability, and problem-free disposal, as well as low health risk influence, so it can be used as a cheap, lightweight reinforcement [10]. For example, energy consumption only amounts to a fifth of the energy necessary for the manufacture of glass-fiber mats [11]. Natural fibers originate from different plant types and plant parts, such as leaf (banana, sisal, and abaca), bast (kenaf, jute, hemp, and flax), fruit (oil palm and coir), seed (milkweed and kapok), grass (bagasse and bamboo), and wood fibers [10], with application as reinforcement in the fields of automotive industries (e.g., interior lining of cars and commercial vehicles), infrastructure sectors, consumer and sports goods [1]. Concurrently, natural fibers such as flax and bamboo have gained attention as reinforcements in composite materials due to their biodegradability, low density, and favorable mechanical properties.

Composites reinforced with natural fibers have been widely used in partial/full replacement as an alternative to synthetic ones [12]. While flax fiber remains one of the most widely studied natural reinforcements due to its high stiffness and tensile strength relative to its low density, bamboo fibers are less frequently reported in polyester-based matrices but are recognized for their high cellulose content and favorable mechanical properties [1]. The combination of bio-based resins with natural fibers presents a promising pathway toward fully sustainable composite materials for automotive, construction, and packaging industries [1]. In parallel, natural fiber-reinforced composites have been extensively studied for their eco-friendly attributes. Among them, flax fibers have been widely studied for their high cellulose content (70–80%) and excellent mechanical properties, making them ideal for lightweight composites [13]. Non-woven flax mats, in particular, could provide uniform fiber distribution and ease of processing in laminate composites. Hybrid composites combining synergistic effects of flax and bamboo fibers with bio-polyester resins have shown enhanced interfacial adhesion and mechanical performance, improving flexural strength and impact resistance compared to single-fiber systems [14,15]. Walter et al. [16] developed two formulations of unsaturated polyester derived from bio-feedstocks, modified with hydroxymethyl methacrylate to achieve workable viscosity. The resin was used in vacuum-assisted resin infusion to produce flax fiber composites exhibiting ~99 MPa tensile strength and a ~9 GPa modulus, with a glass transition temperature of ~70 °C at ~40% fiber volume fraction. Elsewhere, formulations based on bio-PET or PLA have also been reinforced with flax or cotton fibers, with fiber contents of 3–30 wt %, leading to enhanced stiffness but often showing reduced toughness due to poor interfacial adhesion—highlighting a common challenge in matrix–natural fiber compatibility [17].

Despite these advancements, challenges remain in optimizing the curing kinetics, fiber–matrix compatibility, and long-term durability of fully bio-based composites. Some studies have reported that bio-resins exhibit slower curing kinetics than their petroleum-based counterparts, which can affect processing efficiency [18]. Additionally, the hydrophilic natural fibers can lead to moisture absorption, potentially compromising interfacial adhesion in fiber-reinforced composites. Its low thermal stability and deprived adhesion characteristics [10,19,20] could be overcome by optimizing the polymer matrix/fiber system of the composite material where they are incorporated. Key challenges persist in fiber–matrix compatibility, as natural fibers often exhibit poor adhesion with hydrophobic resins. Surface treatments such as alkali treatment, silane coupling, and acetylation have been explored to enhance interfacial bonding (improvement of the surface roughness, reduction in surface -OH groups, or fiber surface functionalization) [21,22]. Additionally, the variability in fiber properties due to natural sourcing necessitates rigorous characterization for consistent composite performance [23].

While previous studies have explored bio-based polyester resins and natural fiber composites independently, there remains a need for systematic investigations into fully bio-based laminate systems with optimized processing and performance. Specifically, most studies focus on woven or short-fiber reinforcements, whereas non-woven flax/bamboo structures, which offer environmental, cost, and processing advantages, are less explored. The shift toward sustainable materials has accelerated research into bio-based polyester resins and natural fiber-reinforced composites, driven by environmental concerns and regulatory pressures. While synthetic fiber composites (e.g., glass or carbon fibers) dominate industrial applications, their high energy footprint and non-biodegradability have spurred interest in fully bio-based alternatives, combining resins derived from renewable resources (e.g., plant oils, lignin) with natural fibers (e.g., flax, bamboo) [24,25,26,27]. However, a critical challenge lies in optimizing the mechanical performance of non-woven natural fiber composites, which typically underperform compared to woven fabric-reinforced systems, so material performances vary significantly depending on fiber architecture. Recent studies suggest that matrix modification through bio-based polyester resins can compensate for these limitations, improving interfacial adhesion and overall composite strength [28,29].

This study aims to contribute to this field by (1) synthesizing a novel bio-based polyester resin from camphoric acid (C_f_A) with improved adhesion properties; (2) partially replacing styrene with another reactive diluent synthesized from natural sources, e.g., trimethylolpropane triacrylate; (3) fabricating laminate composites using non-woven flax and non-woven bamboo mats, primarily used as simple domestic items, as composite reinforcement; and (4) evaluating their mechanical, thermal and morphological properties of synthesized resins and produced composites. This work fills a gap in the literature by examining the performance of bio-based polyester resins combined with natural non-woven and untreated bamboo and flax fibers, rather than more traditional combinations of polyester and woven fabrics. The findings are expected to provide insights into the feasibility of these sustainable composites for structural and semi-structural applications, offering a viable alternative to conventional petroleum-based materials.

## 2. Materials and Methods

### 2.1. Materials

**Figure 1:**
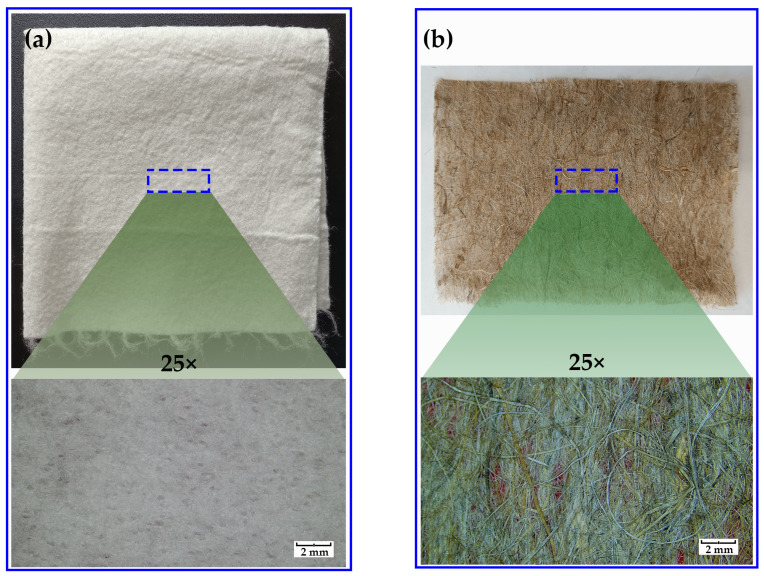
Appearance of (**a**) non-woven bamboo fabric and (**b**) non-woven flax fabric, used as reinforcement in polymer composites, with magnification of 25×.

All chemicals used for the synthesis of unsaturated polyester resins from camphoric acid, non-woven bamboo fabric (nwBf), and non-woven flax fabric (nwFf) with randomly oriented fibers, for the fabrication of composites, are listed in Supplementary Materials S1.1 (SM S1.1) and Table S1. The appearance of the bamboo and flax fabrics is presented in Figure 1. Other bio-based reactants, such as propylene glycol (PG), glycerol (Gly), maleic anhydride (MA), and trimethylolpropane triacrylate (TMPTA), were synthesized according to the procedure given in the literature [29].

### 2.2. Camphoric Acid Preparation

A detailed description of the experimental method is given in Supplementary Materials S1.2 [30]. The product was characterized by acid value (AV) determination, where AV = 550 mg KOH/g (purity 98.1%). The structure of the synthesized C_f_A was confirmed by the results from FTIR and NMR analyses.

### 2.3. Synthesis of Camphoric-Based Unsaturated Polyester Resins (C_f_-UPRs)

A detailed procedure for the synthesis of camphoric acid-based, bio-based, unsaturated polyester resin was presented in our recent work [31] and can be found in Supplementary Materials S1.3 [29,32,33,34,35,36,37]. An additional novelty in the synthesis of the bio-based unsaturated polyester resin was the use of glycerol as a bio-based and sustainable alcohol. Moreover, the introduction of glycerol units is expected to enhance the flexibility of polymer chains, improve fiber/polymer matrix adhesion, and promote better fiber wetting, thereby contributing to more efficient stress transfer and overall composite performance. At 90 °C, the obtained camphoric acid-based unsaturated polyester resin (C_f_-UPR) was dissolved in styrene STY and TMPTA in different mass ratios, so the final dilutions were set at

- 40 wt.% of styrene, and the bio-resin was denoted as C_f_-UPR/STY;
- 20 wt.% of styrene + 20 wt.% of TMPTA, bio-resin was denoted as C_f_-UPR/TMPTA20;
- 10 wt.% of styrene + 30 wt.% of TMPTA, bio-resin was denoted as C_f_-UPR/TMPTA30;
- 40 wt.% of TMPTA, and the bio-resin was denoted as C_f_-UPR/TMPTA.

It was shown from the results of short optimization of C_f_-UPR synthesis with respect to the molar ratio of C_f_A:MA at 1:1.3, 1.1:1.3, 1.2:1.3, and 1:1, that a 1.3:1 molar ratio of MA to C_f_A showed balanced characteristics and was therefore used [31]. Additionally, here, optimization of the Gly:PG ratio at 1:4, 1:5, and 1:6 showed that 1:5 is the optimal for achieving mechanical and fiber wetting properties, processability, and compatibility. Obtained polyesters were further physico-chemically characterized (Table S2) and analyzed by using FTIR, NMR, and TG methods.

### 2.4. Composite Material Production

In order to verify bio-resin properties, neat bio-resins were controllably homogenized for 12 min in a laboratory planetary mixer, designed for vacuum evacuation of gaseous bubbles and any low-boiling material, for appropriate homogenization. The initiator (0.15 wt.% cobalt octoate (CoAc) activator and 1.5 wt.% methyl ethyl ketone peroxide (MEKP)) was added, and the mixture was homogenized at 200 rpm for 2 min under vacuum to remove gas bubbles and produce a homogenous mixture that was immediately poured into polytetrafluoroethylene (PTFE) molds. The C_f_-UPRs were cured at room temperature for 24 h before being placed in an oven at 70 °C for 8 h [31], while the post-curing temperature optimization was necessary. Appropriate post-curing temperature for newly synthesized bio-resins was investigated in order to achieve the desired thermo-mechanical characteristics of the cured materials and following composites (SM S1.4), and it turned out that the optimum post-curing temperature at 100 and 110 °C for 1 h is suitable for C_f_-UPR/STY and C_f_-UPR/TMPTA30, respectively.

For the fabrication of composites reinforced with bamboo and flax non-woven mats, all resin systems described in Section 2.3 were initially considered. Based on the obtained results, C_f_-UPR/STY and C_f_-UPR/TMPTA30 systems were selected for subsequent comparison of the thermo-mechanical properties of composites prepared with resins containing different reactive diluents. C_f_-UPR/STY and C_f_-UPR/TMPTA30, separately, with an initiator, was applied manually by brush over the mat layers in a rectangular PTFE molds (size 180 × 100 × 4 mm), at a composite composition of 70 wt.% of polyester matrix and 30 wt.% of non-woven reinforcement (6 layers or 12.3 g of bamboo mat; 10 layers or 17.1 g of flax mat), leading to a layered composite structure. Previously, non-treated bamboo and flax mats were dried at 70 °C for 6 h to eliminate moisture. A stepwise procedure was performed by applying the resin with immediate degassing of bubbles using an aluminum roller. At the end of this procedure, the vacuum bagging technique was applied and enabled curing without cavities in the material, in the curing and post-curing conditions previously described. Finally, depending on the used bio-resin as polymer matrix and the type of non-woven used as reinforcement, the following materials were obtained:

- Bio-composites with polymer matrix C_f_-UPR/STY, reinforced with a bamboo mat and flax mat, separately, denoted as C_f_-UPR/STY-BAM and C_f_-UPR/STY-FLAX, respectively;
- Bio-composites with polymer matrix C_f_-UPR/TMPTA30, reinforced with bamboo mat and flax mat, separately, denoted as C_f_-UPR/TMPTA30-BAM and C_f_-UPR/TMPTA30-FLAX, respectively.

### 2.5. Characterization Methods

Characterization methods, including testing conditions and equipment, are given in Supplementary Materials S1.5 [38,39,40,41,42,43,44].

## 3. Results and Discussion

### 3.1. FTIR and NMR Analysis of Raw Materials, Bio-Resins, and Composites

**Figure 2:**
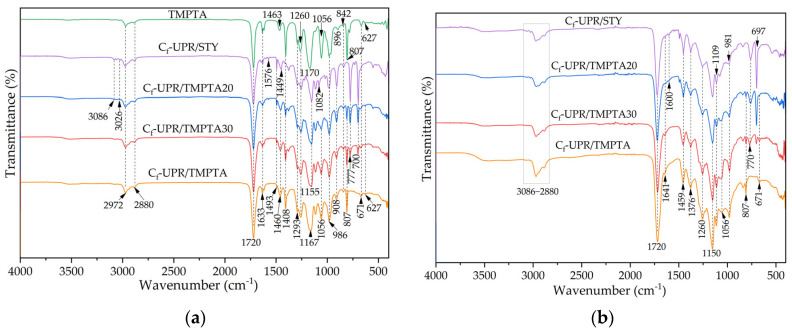

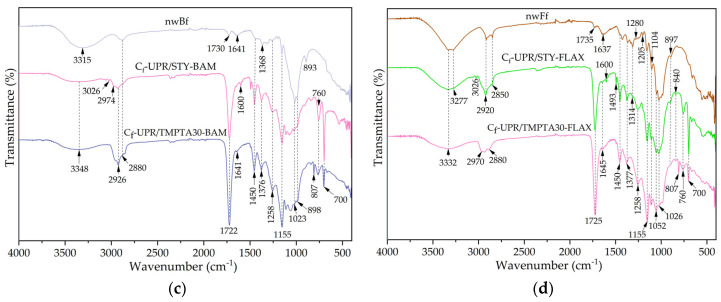
FTIR spectra of synthesized (**a**) C_f_-UPRs, (**b**) cured neat C_f_-UPRs, (**c**) composites with nwBf reinforcement, and (**d**) composites with nwFf reinforcement.

FTIR spectra of raw materials, synthesized camphoric acid-based unsaturated polyester resins (C_f_-UPR), and prepared composite materials are shown in Figure 2.

Detailed characterization of the synthesized camphoric acid and derived resin with styrene reactive diluent was presented in our recent work [31]. For all camphoric resin spectra (Figure 2a) and corresponding cross-linked resin (Figure 2b), the characteristic vibrations of the unsaturated =CH, aliphatic (methylene, -CH_2_, methyl, and -CH_3_), and ester groups can be seen at 3086–3026 and 2972–2880 cm^−1^ regions, as well as at 1720 cm^−1^, respectively. Additionally, all spectra exhibit a small peak (Figure 2a), related to the C=C-stretching vibration, at 1633 cm^−1^ due to the presence of maleic anhydride in the structure of the synthesized resins and used reactive diluent styrene and TMPTA. The peak intensity is related to the type and contents of the used reactive diluent and increases with the increasing quantity of TMPTA. The stretching and bending vibrations at 1576 cm^−1^ and at 1493 and 1449 cm^−1^, respectively, attributed to ethylenic groups vibrations in the spectrum of C_f_-UPR/STY, disappeared with decreasing STY content in the resins. Further, the vibrations between 1260 and 908 cm^−1^ are attributed to C-O-C and C-O stretching bonds in alcohols or esters. The intense band at 807 cm^−1^, observed in the spectrum of TMPTA reactive diluent, and two intense bands at 777 cm^−1^ and 700 cm^−1^ in the spectra of resin with 40% of styrene (C_f_-UPR/STY, Figure 2a), reflect the presence of the =C-H deformation vibration of the vinyl bonds. With the increase in TMPTA content from 20 to 40%, band intensity at 807 cm^−1^ simultaneously increases, while the vibrations from unsaturated bands in the styrene structure decrease and disappear in the spectrum of C_f_-UPR/TMPTA (Figure 2a). Further, due to resin’s cross-linking, the peak shape and intensity related to unsaturated bonds are changed, and a slight shift in characteristic stretching, bending, and out-of-plane deformation peaks for unsaturated C=C and =C-H functional groups in spectra (Figure 2b). It is observed that the same applies to stretching vibrations of C-O and C-O-C functional groups (Figure 2b). Cross-linked resins followed the same pattern as crude resin: bands increased at 1641 cm^−1^, 1459 cm^−1^, and 807 cm^−1^, bands decreased at 1600 cm^−1^, 770 cm^−1^, and 697 cm^−1^, contributing to the disappearance in the spectrum of C_f_-UPR/TMPTA with increasing quantity of TMPTA, and the appearance of a new vibration at 1376 cm^−1^ was attributed to the aliphatic methyl groups.

Figure 2c,d presents the FTIR spectra of flax and bamboo-based composite materials. For all flax- and bamboo-produced materials, spectra mostly show peaks from resins (Figure 2c,d). The flax spectrum shows peaks related to the functional groups found in the structure of hemicellulose, cellulose, and lignin. Vibrations at 1735 cm^−1^, 1633 cm^−1^, 2920–2850 cm^−1^, as well as 1400–1315 cm^−1^ and 1377 cm^−1^, are due to stretching vibrations of C=O in hemicelluloses, bending vibrations of O-H in cellulose, -CH stretching and -CH bending vibrations in all structures; the peak at 1377 cm^−1^ relates to the vibrations of methoxy group from syringyl and guaiacyl units in lignin [31,45]. The vibrations of C-O/C-OH bands of the glycosidic ring and *β*(1–4) glycosidic bonds are observed at the 1280–980 cm^−1^ region and the 897 cm^−1^ region. In the comparison spectra between flax fabric and the produced composite, bands around 3332 and 3277 cm^−1^, as well as around 1104–1026 cm^−1^, assigned to the O-H hydroxyl groups and C-O/C-O-C from the flax, were present in spectra C_f_-UPR/STY-FLAX, and C_f_-UPR/TMPTA30-FLAX. The other, as a result of the interaction of resins with flax, shifted the band for C=O bonds to 1725 cm^−1^. The same applications can be seen for peaks at 1645 and 760–700 cm^−1^, while the peak at 807 cm^−1^ in spectrum C_f_-UPR/TMPTA30-FLAX decreases. The spectra of bamboo non-woven (Figure 2d) show the following bands at 3315 cm^−1^ (O-H stretching), 1730 cm^−1^ (C=O stretching), 1641 cm^−1^ (O-H-O bending), 1450–1315 cm^−1^, 1368 cm^−1^ (syringyl and guaiacyl units from lignin), 1258–898 cm^−1^ (C-O/C-O-C/O-C=O stretching), and 893 cm^−1^ (glycosidic bond vibration) [46]. The new bands observed in the spectra of C_f_-UPR/STY-BAM and C_f_-UPR/TMPTA30-BAM composites (Figure 2d), found at 1023–898 cm^−1^, around 3000, and at 2880 cm^−1^, originate from the surface functional groups present in bamboo reinforcement. The change in band structure can be attributed to the successful cross-linking of unsaturated resins and intermolecular interactions of the matrix with the bamboo fabric.

**Figure 3:**
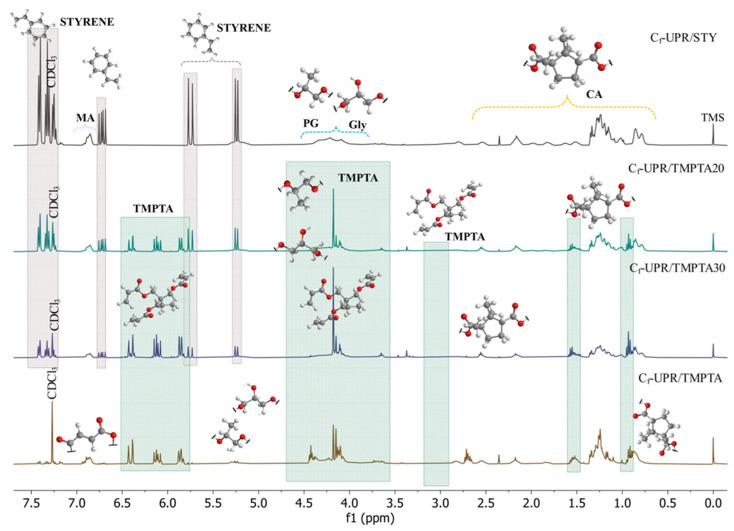
^1^H NMR spectra of camphoric acid-based unsaturated resin C_f_-UPR/STY, C_f_-UPR/TMPTA20, C_f_-UPR/TMPTA30, and C_f_-UPR/TMPTA.

**Figure 4:**
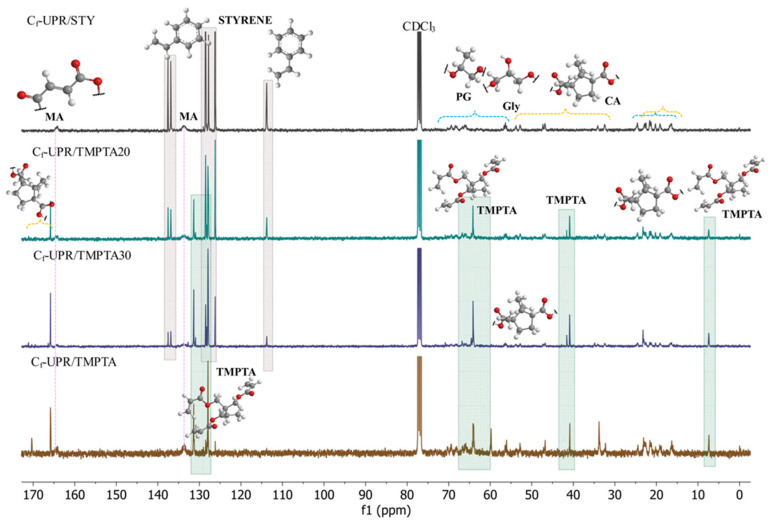
^13^C NMR spectra of camphoric acid-based unsaturated resin C_f_-UPR/STY, C_f_-UPR/TMPTA20, C_f_-UPR/TMPTA30, and C_f_-UPR/TMPTA.

Further characterization of the unsaturated resins based on camphoric acid C_f_-UPR/STY, C_f_-UPR/TMPTA20, C_f_-UPR/TMPTA30, and C_f_-UPR/TMPTA was conducted through ^1^H and ^13^C NMR spectroscopy, as displayed in Figure 3 and Figure 4. The NMR spectrum of the TMPTA is shown in Supplementary Materials S2.1 [47], Figure S2, while illustrative NMR spectra with detailed characterization of a camphoric acid are shown in previous work [31] (Figures S3 and S4). It was explained that the NMR spectra (Figure 3 and Figure 4) of all unsaturated resins used show complex structures as a result of the stereochemistry of camphoric acid. The atom numbering of the four main possible convenient structures due to the stereochemistry of camphoric acid is given in Figure S5.

^1^H NMR spectra displayed characteristic signals from camphoric acid [31], such as chemical shifts at 0.79–0.86 ppm region, assigned to (C(6)-H_3_, C(7)-H_3_, and C(8)-H_3_); at 1.51–2.54 ppm, assigned to (C(3)-H_2_ and C(4)-H_2_); and 2.81–2.94 ppm, assigned to (C(2)-H). All of them relate to methyl and methylene protons, respectively. Additionally, signals at 1.17–1.35 in the polyester resin were attributed to the methyl protons of propylene glycol (PG), while methylene protons of the propylene glycol and glycerol (Gly) moieties appeared in the 4.09–4.33 ppm region. The signals at 6.85–6.89 ppm were assigned to the protons of the maleic anhydride moiety, i.e., C(14)-H and C(15)-H hydrogen. Also, the multiple peaks observed at 5.12–5.30 are observed and assigned to C(18)-H and C(19)-H in Structures I, II, III, and IV (Figure S5), which are overall assigned to vinyl protons from the styrene (5.23–5.26 ppm) moiety in C_f_-UPR/STY, C_f_-UPR/TMPTA20, and C_f_-UPR/TMPTA30 spectra. With the increase in TMPTA content in relation to STY, the intensity of the signal’s changes, and the peaks at 5.23–5.26 from styrene decrease. The doublet of doublet at 6.72, multiplet at 7.23–7.24, triplet at 7.32, and doublet at 7.41 ppm, observed in spectra of resin with 10, 20, and 40% of styrene, are assigned to the =CH groups of the vinyl group and phenyl ring of styrene, respectively. As shown in Figure 3, resins with 20, 30, and 40% of TMPTA exhibited additional new signals 0.87–1.15, 1.45–1.60, 5.82, and 6.47 ppm, corresponding to methyl, methylene, and vinyl protons, respectively, which resulted from the presence of TMPTA (Figure 3 and Figure S2). Moreover, the significant changes in the proton signals, which are associated with alcohol units (PG and Gly), and the appearance of new signals, between 2.50 and 4.44 ppm, further confirmed the successful incorporation of TMPTA and monomer moieties in the resin structure.

The ^13^C NMR spectra of all unsaturated resins (Figure 4) showed signals at 16.4–22.9, 24.6, 32.5–34.2, 46.9–47.3, 52.9–53.8, and 56.1–56.7 ppm, corresponding to carbons from camphoric acid and alcohol moieties (C(6), C(7), C(8), C(11), C(12), C(22), C(23), C(3), C(4), C(1), C(2), and C(5)) which is in good agreement with previous work [31]. Further, the signals observed at 65.8–69.3 ppm were assigned to the carbon C(10) (Structures I and II), and C(12), (Structures III and IV), C(17), C(18), and C(19) (Structures I–IV), corresponding to the alkyl skeleton of the PG and Gly units (Figure S5). The vinyl carbons and carboxyl carbon, from the maleic anhydride moiety, were observed at 133.9 and 164.1 ppm, respectively [31], while peaks at 113.7–113.9, 126.2–129.0, and 136.8–137.5 ppm were ascribed to vinyl and aromatic carbons in styrene (Figure 4). The ester carbonyl C(9) and C(20) from the camphoric acid moiety appeared at 173.4 ppm (Figure 4) [31]. The spectra of polyester resins with TMPTA exhibited new signals at 7.4 ppm and 40.8–40.9 ppm, as well as at 59.8–64.3 ppm, corresponding to methylene carbons from TMPTA (Supplementary Materials S2.1, Figure S2). In addition, peaks at 130.9–131.3 ppm, as well as between 127.8 and 128.3 ppm, were assigned to the vinyl carbon in the TMPTA structure (Supplementary Materials S2.1, Figure S2). Due to the stereoisomerism present in the camphoric acid structure (Figure S4), the esterified resins, which are cross-linked with TMPTA, showed multiple ester carbonyl group signals for C(9) and C(20) observed at 166.9–170.0 ppm in C_f_-UPR/TMPTA20, C_f_-UPR/TMPTA30, and C_f_-UPR/TMPTA spectra (Figure 4).

### 3.2. Rheological Behavior of Uncured Resins

**Figure 5:**
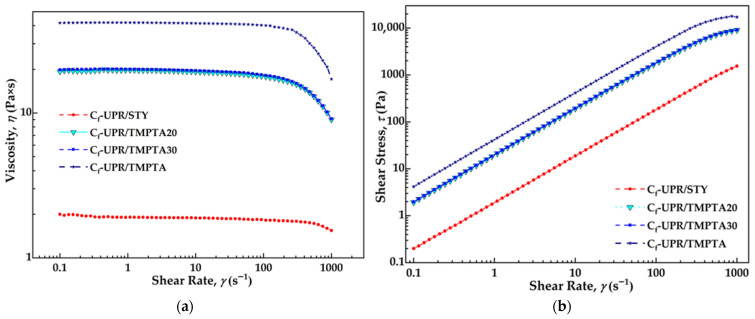
Effect of TMPTA reactive diluent on C_f_-UPR viscosity: (**a**) shear rate dependence of the viscosity and (**b**) shear rate dependencies of the shear stress for C_f_-UPR/STY, C_f_-UPR/TMPTA20, C_f_-UPR/TMPTA30, and C_f_-UPR/TMPTA.

A rheological study of synthesized unsaturated polyester resins, C_f_-UPR/STY, C_f_-UPR/TMPTA20, C_f_-UPR/TMPTA30, and C_f_-UPR/TMPTA, was performed to provide information about how the shear rate, as a processing condition, affects the fluid behavior (Supplementary Materials S1.5). Rheograms of the examined resins, shear rate dependence of the viscosity, along with the shear rate dependence of the shear stress, measured isothermally at 25 °C, are shown in Figure 5. The viscosity of the C_f_-UPRs was found to be independent up to the shear rate of 100 s^−1^, which is characteristic of a Newtonian liquid, and it indicates an absence of chain entanglements.

As seen in the graph in Figure 5a, C_f_-UPR/STY, with styrene as reactive diluent, showed almost constant viscosity of around 1.9 Pa·s within the range from 0.1 to 100 cm^−1^ of the applied shear rate. The viscosity of the resin increased with an increasing amount of TMPTA as a replacement for styrene reactive diluent, as is shown in the literature [8]. This could be a result of the higher viscosity of TMPTA itself, compared to the styrene, as well as a higher polarity of TMPTA. In general, the molecules of the reactive diluent penetrate between the polyester molecules and separate them, thus decreasing the intermolecular interactions between them [48]. In this case, non-polar styrene molecules caused a greater decrease in intermolecular interactions between the larger and polar resin molecules than in the case of polar TMPTA molecules. However, taking into account the obtained viscosity values for C_f_-UPR with mixed STY and TMPTA diluents (from 19.3 to 22.6 Pa·s), all the resins could be used in the suitable process of composite production. Moreover, a linear relationship between shear stress and shear rate was observed for all resin samples, as depicted in Figure 5b, which reflects the Newtonian fluid behavior of C_f_-UPR/STY, C_f_-UPR/TMPTA20, C_f_-UPR/TMPTA30, and C_f_-UPR/TMPTA [43]. In all cases, the shear stress increased with the shear rate, because a higher force would be required to deform the intermolecular forces among the resin molecules.

### 3.3. Water Absorption

The water absorption rates of the cured C_f_-UPR/STY and C_f_-UPR/TMPTA30 resins and fabricated composites are summarized in Table 1 and Figure S6. The neat cured resins exhibit negligible water absorption, measured at only 0.21% and 0.23% for C_f_-UPR/STY and C_f_-UPR/TMPTA30 after 24h, respectively, which remains constant throughout the observation period.

A significant increase in water uptake, in comparison with cured neat resins such as C_f_-UPR/STY and C_f_-UPR/TMPTA30, is observed with laminated composites with natural fiber reinforcement. Abundant hydroxyl groups within cellulose and hemicellulose, apropos natural fiber hydrophilic nature, had an influence on the higher water absorption observed. In addition, the porous nature of natural fibers could affect water diffusion within the structure. However, in the case of the polymer composites C_f_-UPR/TMPTA30-BAM and C_f_-UPR/TMPTA30-FLAX, decreased water absorption can be observed, which is probably associated with improved interfacial bonding between the polymer matrix and fibers, as well as higher cross-linking density, compared to composites based on C_f_-UPR with a styrene reactive diluent. In addition, chemical modifications of natural fabrics and fibers may be applied to enhance the compatibility between the hydrophilic fibers and hydrophobic UPR, leading to improved water absorption performance, i.e., water repellency, and application of hybrid composites in humid environments [49,50].

### 3.4. Mechanical and Dynamic-Mechanical Properties of Cured Neat C_f_-UPRs and Composites

#### 3.4.1. Tensile and Flexural Properties

**Figure 6:**
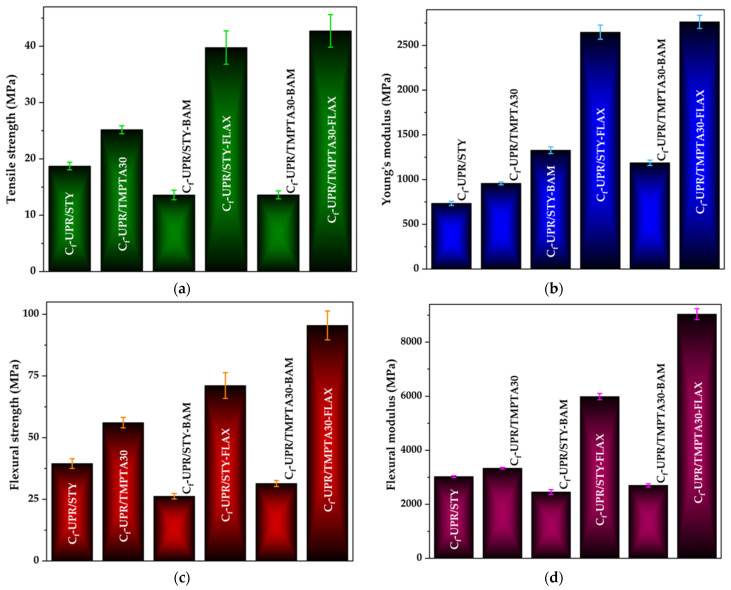
Tensile and flexural properties for neat C_f_-UPR/STY and C_f_-UPR/TMPTA30 matrix and fabricated composites: (**a**) tensile strength, (**b**) Young’s modulus, (**c**) flexural strength, and (**d**) flexural modulus.

For the prepared polymer composites with the ratio C_f_-UPR polymer matrix to natural reinforcement (7:3), obtained tensile strength and flexural modulus are presented in Figure 6.

Tensile testing revealed distinct effects of polymer matrix formulation and non-woven reinforcement on mechanical performance. Among the neat C_f_-UPRs, the C_f_-UPR/TMPTA30 exhibited higher tensile strength (Figure 6a) and Young’s modulus (Figure 6b) compared with the C_f_-UPR/STY, suggesting that substitution of STY with TMPTA altered the network architecture. These results revealed that the bio-based polyester resin C_f_-UPR/TMPTA30, with the introduction of a multifunctional acrylate TMPTA, which typically raises the effective cross-link density and network connectivity of the polyester matrix, exhibited both higher tensile strength and an increased Young’s modulus, suggesting a sustainable material for higher stresses and with greater stiffness under tensile loading.

When reinforced with nwBf, both composite materials showed lower tensile strength and Young’s modulus than the corresponding neat resins. Importantly, this underperformance cannot be attributed to poor fiber–matrix adhesion, which was found to be adequate; rather, it is most plausibly explained by the fabric architecture and processing effects. The bamboo reinforcements were supplied as non-woven mats, which tend to produce random fiber orientations, lower effective fiber volume fraction in the load direction, resin-rich zones, and potentially higher porosity or poor consolidation during lamination. Such structural and processing shortcomings reduce the efficiency of load transfer from the polymer matrix to fibers and create stress concentrators, thereby negating the benefit of good interfacial bonding and resulting in composites that are weaker and less stiff than the neat matrices. In addition, the increase in lignin content in nwBf, according to the results from Table S3, is associated with a decline in the mechanical performance of bamboo reinforced composites C_f_-UPR/STY-BAM and C_f_-UPR/TMPTA30-BAM.

In contrast, nwFf-reinforced composites exhibited a marked improvement in both tensile strength and Young’s modulus relative to the neat resins, with the combination of flax mats and the C_f_-UPR/TMPTA30 delivering the best overall performance (Figure 6a,b). This enhancement points to a more effective reinforcement action by the nwFf, which may arise from a combination of higher intrinsic fiber stiffness/strength, a more favorable fabric architecture or fiber length (higher local fiber packing and orientation in the load direction), and superior polymer matrix/fiber interaction in this particular resin/fiber pairing. The superior performance of the C_f_-UPR/TMPTA30 when coupled with flax further suggests a synergistic effect between matrix chemistry and fiber type that improves stress transfer and delays failure.

Overall, these results indicate that, beyond intrinsic matrix properties, fabric architecture (non-woven vs. more consolidated weave), fiber volume fraction, and processing quality (impregnation, consolidation, and void content) are critical determinants of composite tensile behavior. To substantiate these interpretations, SEM fractography characterization is recommended. Scanning electron microscopy (see Section 3.5) indicated a good interfacial adhesion between the C_f_-UPR/TMPTA30 and nwFf reinforcement, which results in the enhancement of composite tensile properties. Besides C_f_-UPR/STY-FLAX, where lower tensile properties were observed, possibly due to the fiber pullout, suggesting a weaker adhesion between the fibers and polymer matrix, C_f_-UPR/TMPTA30-FLAX exhibited improved tensile properties values, as can be observed in SEM micrographs with fiber fracture as a result of better C_f_-UPR/TMPTA30 and fiber adhesion, resulting in long-fiber-reinforcing effectiveness.

Figure 6c,d shows the flexural strength and modulus, respectively, obtained from the three-point bending test for the examined cured neat C_f_-UPR/STY and C_f_-UPR/TMPTA30, along with fabricated composites. The initial load vs. displacement behavior appeared linear for all cases (Figure S7), which indicates stable behavior until the initiation of test specimen failure occurred immediately before the maximum load point was reached. Beyond the maximum load point, the load decreased for the obtained composite specimens, thus indicating delamination and/or composite crack propagation. The neat cured C_f_-UPR/STY and C_f_-UPR/TMPTA30 showed an abrupt load decrease, in contrast to the nwBf- and nwFf-reinforced composites.

C_f_-UPR/TMPTA30 had the better flexural performance, such as flexural strength (56.13 MPa) and flexural modulus (3.338 GPa), with an improvement of 42% and 10%, respectively, in comparison to the C_f_-UPR/STY flexural strength and flexural modulus values. A significant positive effect of the incorporation of TMPTA into polyester resin was observed as results increased in flexural properties, observed for the nwFf-reinforced composite, C_f_-UPR/TMPTA30-FLAX, with a 70.1% increase compared to the pure polymer matrix. Flexural strength and modulus of C_f_-UPR/TMPTA30-FLAX were observed to be 95.49 MPa and 9.039 GPa, respectively, which were higher among all other obtained composites, revealing the strong interfacial adhesion between fabrics and matrix.

**Figure 7:**
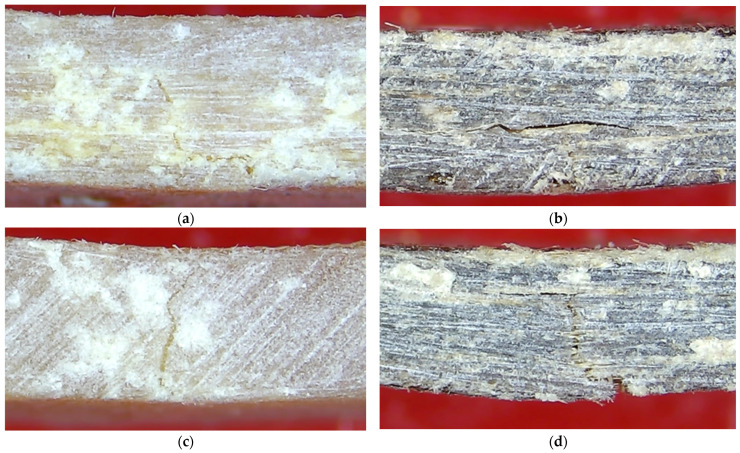
C_f_-UPR/STY-based composites with (**a**) nwBf and (**b**) nwFf, as well as C_f_-UPR/TMPTA30-based composites with (**c**) nwBf and (**d**) nwFf, after being subjected to three-point bending test.

A remarkable difference between C_f_-UPR/STY and C_f_-UPR/TMPTA30-based composites is shown in Figure 7. The fracture surfaces of the tested composite specimens were imaged using an optical microscope (SMTV Visor Inspection System, Michael Bruch, Germany) after three-point band test failure. Figure 7a,b indicates that the initiation and evolution of cracks is the dominant damage mechanism in the case of the composites based on C_f_-UPR/STY, so adhesive failure can be observed due to the unsteady delamination of natural fibers, bamboo and flax, from the C_f_-UPR/STY (fiber–matrix failure), indicating weaker interfacial bonding between the fiber and the polymer matrix. Contrarily, because of the improved wettability of nwBf and nwFf with C_f_-UPR/TMPTA30, cross-section failure of C_f_-UPR/TMPTA30-BAM, especially for C_f_-UPR/TMPTA30-FLAX specimens, is obvious due to the improved fiber/polymer matrix adhesion, as seen in Figure 7c,d. It is plausible that the excellent reinforcing and stiffening effect of long nwFf, satisfying adhesion of polymer matrix to fibers, as well as the physical interaction between the fibers and polymer matrix, may jointly contribute to an improvement in resilience of the fabricated composites. These results of tensile and flexural properties are also supported by DMA and SEM.

In order to compare the mechanical properties of bio-based UPRs and obtained composites with natural fiber reinforcement, Table 2 gives the values of tensile strength, modulus of elasticity (Young’s modulus), flexural strength, and flexural modulus from the limited literature for non-woven reinforced UPR composites.

Most studies reported that non-woven natural fibers, particularly flax and bamboo, provide only moderate reinforcement to UPR compared to woven or unidirectional fabrics. In bamboo-based UPR composites, tensile strengths around 15–25 MPa and flexural strengths below 50 MPa were typical, mainly due to weak fiber–polymer matrix adhesion and irregular fiber distribution. Non-woven flax mats performed slightly better, with tensile strengths between 30 and 40 MPa, yet still lower than aligned flax laminates. Researchers often attributed the lower mechanical properties to void content, poor resin impregnation, and random fiber orientation. Alkali or silane surface treatments improved tensile and flexural moduli by enhancing bonding at the interface. However, even after treatment, the elastic modulus values (2–4 GPa) remained significantly below those of synthetic-fiber composites. Studies emphasized that UPR’s brittleness and limited compatibility with hydrophilic fibers further restrict stress transfer efficiency. Overall, while non-woven bamboo and flax mats are promising eco-friendly reinforcements, their mechanical performance remains modest unless combined with chemical modification or hybridization strategies.

#### 3.4.2. Dynamic-Mechanical Properties

Dynamic-mechanical analysis (DMA) is one of the effective ways to evaluate viscoelastic properties and interfacial features of the fiber-reinforced polymer system. The dynamic-mechanical behavior of the composites depends on their thermo-mechanical history, type and architecture of the reinforcement, and fiber content, among others [54], and provides molecular-level information to understand the mechanical properties of materials [55,56]. Prior to DMA, all samples were subjected to an amplitude sweep test in order to determine the linear viscoelastic region (LVR), i.e., the range within which the material exhibits linear behavior and the deformations remain independent of the applied stress. Subsequently, frequency sweep tests were performed to examine the dependence of storage and loss moduli on frequency, thereby providing insight into the relaxation processes and time-dependent behavior of the materials. These preliminary tests ensure a reliable basis for the interpretation of the main DMA results, as described in Supplementary Materials S2.3 [57].

**Figure 8:**
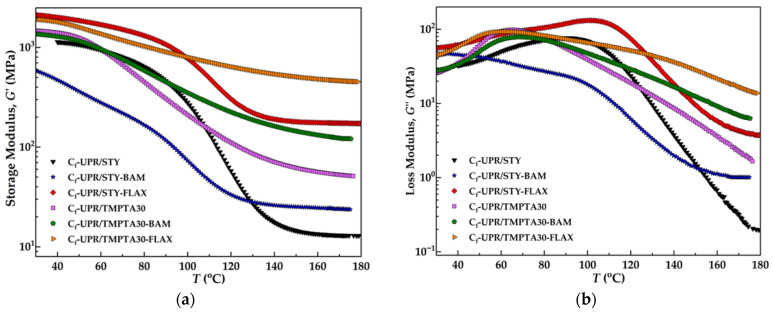

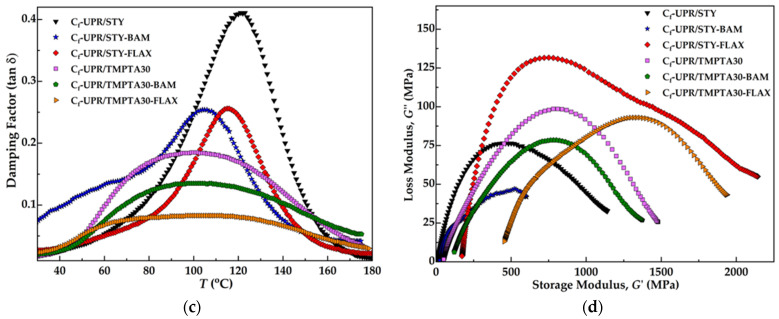
Dynamic-mechanical properties for neat C_f_-UPR/STY and C_f_-UPR/TMPTA30 matrices and fabricated polymer composites: (**a**) storage modulus, (**b**) loss modulus, (**c**) damping factor, and (**d**) Cole–Cole analysis.

The storage modulus (*G*′) represents the stored energy in the material’s elastic structure and corresponds to its elastic response, and in other words, reflects the energy recovered when the stress is released, a measure of deformation energy stored by the composite material during the shear process, thus showing completely reversible deformation behavior [58,59]. This is particularly beneficial to evaluate the stiffness and elastic behavior of the fabricated materials. The variation in the storage modulus with respect to the temperature for the fabricated composites is illustrated in Figure 8a.

The temperature range is set so that *G*′ curve reaches a plateau. As the temperature rises and approaches the glass transition temperature, *G*′ decreases gently, indicating the transition from a glassy to a rubbery state [60]. After the glass transition temperature, a decline in the storage modulus is evident for all samples. The slight decrease in storage moduli in the rubbery plateau for composites reinforced with nwBf and nwFf reflects the weakening of the secondary bonds between the polymer matrix and the fibers, higher mobility of the fibers, and the higher free volume between polymer chains, so the viscoelastic behavior of the entire system changes [61].

On the other hand, the fabricated composites, also known as materials with natural flax fiber reinforcement, demonstrate higher initial storage moduli, enhancing the stiffness of the material, except for the composites with bamboo fiber reinforcement, C_f_-UPR/STY-BAM and C_f_-UPR/TMPTA30-BAM, thus having a lower initial storage modulus in comparison with the pure polymer matrix. This observation is correspondent with the results of tensile properties (Figure 6). In particular, the C_f_-UPR/STY-FLAX and C_f_-UPR/TMPTA30-FLAX composites showed a more substantial increment of initial storage modulus at 40 °C, from 1.14 GPa (C_f_-UPR/STY) and 1.41 GPa (C_f_-UPR/TMPTA30) to 2.00 GPa and 1.81 GPa, respectively. It appeared that the nwFf reinforcement in C_f_-UPR/STY-FLAX and C_f_-UPR/TMPTA30-FLAX composites had a positive effect on the composites’ elasticity, as revealed by the increase in storage modulus as compared to neat C_f_-UPRs. The high storage modulus indicates that the flax fibers primarily contribute to the enhanced elastic property of the obtained composites. Fibers promote an efficient stress transfer between them and the polyester matrix, creating a positive effect on the elasticity [62,63]. An increase in storage modulus is noted because the fibers have elastic behavior—no energy dissipation from fibers with an increase in temperature or stress is imposed, and because the fiber/polymer matrix interface transfers the stress imposed to the composites from the polymer matrix through the fibers, the energy dissipates as heat is observed by increasing the molecular mobility of the polymer [54]. In addition, the higher the reinforcement ratio, the higher the molecular restriction in the polymeric chains imposed by the reinforcement [64,65]. At a temperature range above 100 °C (for C_f_-UPR/STY-based composites) and above 60 °C (for C_f_-UPR/TMPTA30-based composites), the storage modulus values dropped to a minimum and remained plateaued, which portrays that the material has reached a rubbery state [66]. In addition, at the glassy state, the stiffness promoted by the fibers was not much improved as at higher temperatures [67]. C_f_-UPR/STY-FLAX and C_f_-UPR/TMPTA30-FLAX exhibit the highest increase in storage modulus in the rubbery plateau region (Table 3), 1228% and 758% in comparison to neat C_f_-UPRs, respectively, and also, with cross-linking density (Table 3), 1311% and 690% in comparison to neat C_f_-UPRs, respectively. Generally, composites containing fiber reinforcement exhibit a higher rubbery modulus since the rigidity of the filler and the filler/polymer matrix interface is less affected by the temperature in comparison to the rigidity of the neat polymer matrix. That leads to a decrease in polymer chain mobility at the filler/polymer matrix interface that is reflected in higher cross-linking density of the composites [68,69]. Hence, in the case of DMA, the entire curve must be analyzed because of the thermo-mechanical history of the polymer, using a specific region to describe the material behavior under a wide range of temperatures [67].

Table 3 shows some characteristic parameters suitable for defining the influence of temperature dependences of storage modulus in glassy, transition, and rubbery regions at 40 °C, 100 °C, and 160 °C. Besides the given temperature values, the glass transition temperature (*T*_g_) is sometimes also derived from the onset of the pronounced decrease in the storage modulus curve; the results are shown in Table 3.

The cross-linking density is derived from the rubbery elasticity theory, which implies the absence of polymer chain entanglements [61], as described in Supplementary Materials S1.5. Numerous studies indicate that heterogeneity and restricted mobility of the polymer system arise from the presence of cross-links within the polymer network [70]. Cross-links decrease the number of possible conformational motions, meaning conformational freedom of polymer chain segments, resulting in limited mobility regions around the cross-link points. From Figure 8a is observed that the storage modulus goes up with flax fiber incorporation due to the hindered mobility of the C_f_-UPR/STY and C_f_-UPR/TMPTA30 chains by fiber fillers [55,71].

Using the storage modulus values, the coefficient of reinforcement (effectiveness coefficient, *C*) could be calculated [72]. The coefficient of reinforcement evaluates the effectiveness of fiber reinforcement in a way that a higher value of *C* means a lower effectiveness of fiber dispersion in the polymer matrix. The coefficient of reinforcement can be calculated by Equation (1) [73].

C = ((G′_GS_/G″_RP_)_composite_)/((G′_GS_/G″_RP_)_UPR_) (1)

The data include temperature dependences of storage modulus in glassy (*G*′_GS_) and rubbery (*G*′_RP_) regions for composite and corresponding polymer matrix, at 40 °C and 160 °C. Table 4 displays the coefficient of reinforcement for the prepared composites C_f_-UPR/STY-BAM, C_f_-UPR/STY-FLAX, C_f_-UPR/TMPTA30-BAM, and C_f_-UPR/TMPTA30-FLAX. A lower value of *C* is observed in the case of composites with nwFf, regardless of polymer matrix, indicating a higher efficiency of flax fiber reinforcement. A drop in the coefficient of reinforcement occurred due to greater polymer chain mobility at higher temperatures, and thus, less rigidity was the consequence [74]. These results follow the ones obtained for cross-linking density value (Table 3), showing that higher restriction of polymer segment movements is noticed for the nwFf-reinforced composites.

Contrary to the storage modulus, the loss modulus, *G*″, is helpful to evaluate the viscous attribute of polymeric materials [75]. The quantification of the *G*″ is made based on the energy that is dissipated as heat when subjected to a load cycle, meaning the energy lost through friction and molecular motions [58,59]. Damping or loss factor (*tan* δ) represents the *G*″/*G*′ ratio, meaning an indication of balance between elastic and viscous phase in a polymeric material, and its corresponding maximum (peak) is used in the literature to compare polymers. The damping factor is extremely sensitive to all kinds of relaxation processes, material structure heterogeneity, and the morphology of heterogeneous systems, such as composite materials [76,77,78]. The values of the damping factor curve peak and glass transition temperature (*T*_g_) from the damping factor curve max of neat cured C_f_-UPR/STY and C_f_-UPR/TMPTA30, and derived composites with nwBf and nwFf reinforcements, are given in Table 5. In the case of C_f_-UPR/STY and fabricated composites, flexible natural fibers participate in overall segment motions, which are reflected in lower *T*_g_ values (plasticizing effect), with present -OH surface groups that participate in the creation of hydrogen/dipole–dipole bonding interactions with polymer matrix. Also, incorporation of TMPTA as a replacement of STY in the case of C_f_-UPR/TMPTA30 lowers the *T*_g_ because of steric hindrance in polyester, reflecting in higher mobility of polymer segments.

Generally, thermosets have high cross-link density, which results in higher *T*_g_, higher modulus, and lower damping factor due to the molecular motion restriction [79,80]. Figure 8c clearly indicates that C_f_-UPR/STY showed the highest *tan* δ peak, indicating a more viscous characteristic with high polymeric chain mobility [81]. Bamboo and flax fiber reinforcements in the polymer matrix have substantially affected the damping property of the composites, making them more elastic than the neat cured polyester, which appears as lowered *tan* δ peaks. Among all the reinforced composites, the C_f_-UPR/STY-FLAX and C_f_-UPR/TMPTA30-FLAX composites showed the most significant *tan* δ reduction compared to the polyesters, C_f_-UPR/STY and C_f_-UPR/TMPTA30, by 37% (from 0.41 to 0.26) and by 50% (from 0.19 to 0.08), respectively.

**Figure 9:**
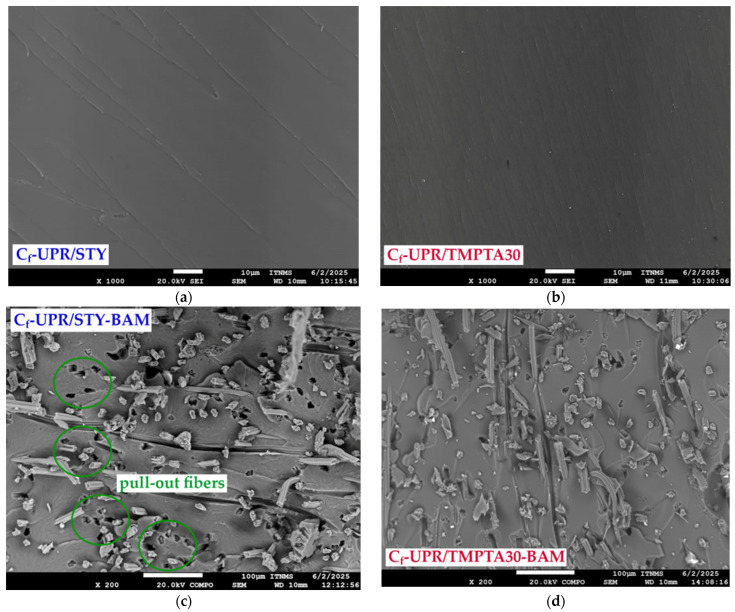

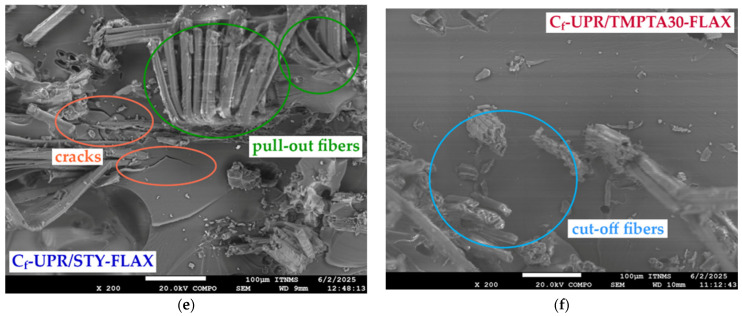
SEM micrographs of the fracture surface of the neat cured (**a**) C_f_-UPR/STY and (**b**) C_f_-UPR/TMPTA30, as well as the fabricated composites (**c**) C_f_-UPR/STY-BAM, (**d**) C_f_-UPR/TMPTA30-BAM, (**e**) C_f_-UPR/STY-FLAX, and (**f**) C_f_-UPR/TMPTA30-FLAX.

A significant change is noted when analyzing the peak width and height, where the peak height decreases with reinforcement, as well as the type of unsaturated polyester resin. So, the fiber allowed less energy to be dissipated through the interface, which is in correlation with the *G*′ and *G*″, and mobility, and the entanglement dynamics of the polymer chains (vibration of the polyester chains is higher) decreased by fiber reinforcement (lower molecular vibration) [67]. Additionally, peak height reduction strongly indicates the polymer chain restriction by an immobilized layer [82], which is an indication of a larger amount of the constrained regions and the existence of fiber/polymer matrix interactions. This phenomenon contributes to the reduction in peak height in the transition zone and consequently a higher storage modulus in the elastomeric and loss modulus in the viscous region [58]. Comparing the values of *tan* δ peaks for C_f_-UPR/STY and C_f_-UPR/TMPTA, it can be concluded that incorporation of TMPTA as a replacement for STY leads to the creation of stronger bonding in the polymer matrix and in the polymer matrix/fiber interface promoting interactions between the fiber and C_f_-UPR/TMPTA30, which is also confirmed by the results of SEM in Figure 9d,f.

With the fiber reinforcement, the amorphous region decreases (referred to the cured neat C_f_-UPRs) as well as the energy dissipation because there are lower molecular chain segments with higher mobility “available”. On the other hand, a high *tan* δ value indicates a greater degree of molecular mobility, so there is more viscous material with high energy dissipation potential, whereas a low *tan* δ is associated with a highly elastic material capable of storing energy within its structure [66]. Moreover, C_f_-UPR/TMPTA30-based composites with nwBf and nwFf reinforcement show an increase in internal friction and additional viscoelastic energy dissipation, which may result in the widening of the transition region [55], although the observed broadening of the transition region can be related to heterogeneity in the molecular weight between the cross-links [68]. The composites with poor interfacial bonding dissipate more energy, producing a high *tan* δ value compared to materials with a strongly bonded interface [77]. The lower *tan* δ value indicates good interfacial adhesion between nwBf/nwFf and the C_f_-UPRs [75,83]. Researchers [84] found that the materials that possess a lower *tan* δ value have shown better load-bearing properties due to improved interfacial adhesion. As the increasing fiber/polymer matrix interface bonding, mobility of the molecular chains decreases, and a reduction in *tan* δ occurs, which shows lower damping, also meaning that the stress transfer between fiber/polymer matrix is increased and fiber/polymer matrix interfacial bonding is also increased [83].

The reduction in the damping factor peak and the broadening of the peak indicate a decrease in the mobility of C_f_-UPR/TMPTA molecules and an improved interface bonding; hence, it can be used to estimate constrained chains [55]. The volume fraction of the constrained region, *C_υ_*, can be obtained by using Equation (2) [85]:

C_υ_ = 1 − (1 − C_0_)⸱W/W_0_ (2)

where *C_υ_* and *W* are the volume fraction of the restricted mobility of polymer chains and the energy fraction loss in the composite, respectively. *C*_0_ and *W*_0_ are the volume fraction of the constrained region and the energy fraction loss for pure polymer matrix, respectively. The energy loss fraction *W* can be calculated by Equation (3) [55].

W = *tan* δ_max_/(*tan* δ_max_ + 1) (3)

Table 4 shows the values of constrained chain volume, and it can be observed that *C_υ_* increases with fiber incorporation. Such an effect is caused by the hindrance of fibers to restrict the polymer matrix [55].

Good adhesion between the polymer matrix and natural fiber reinforcement is important for the composites, especially at temperatures above the glass transition temperature [86]. The adhesion efficiency of fiber/polymer matrix can be calculated by the adhesion factor *A*, which is shown in Equation (4) [87]:

A = 1/(1 − V_f_)⸱(*tan* δ_com_/*tan* δ_UPR_) − 1 (4)

where *tan* δ_com_ and *tan* δ_UPR_ are the relative damping ratios of the composite and the polymer matrix (damping factor peak height), respectively, and *V*_f_ is the volume fraction of the fibers (from Table 4). The lower value of *A* indicates a better interaction at the polymer matrix–fiber interface, but the negative value of *A* is due to the anisotropy of the fiber and improvement of the interface region of the composite [87]. Adhesion factors from Table 5 indicate significantly lower values for nwFf-reinforced composites, especially in the case of a composite based on C_f_-UPR/TMPTA30 with TMPTA as a replacement for the STY reactive diluent, meaning better interaction at the polymer matrix–fiber interface, which is also in correlation with better stiffness and a higher storage modulus.

The *tan* δ curves of the C_f_-UPR/STY and C_f_-UPR/TMPTA30 show that incorporation of TMPTA as reactive diluent leads to a shift in the glass transition to a lower temperature while broadening the *tan* δ peak. This suggests that UPR with TMPTA may introduce more complex network structures within the resin, leading to increased energy dissipation and affecting the composite’s thermal behavior. As expected, the transition regions in the case of C_f_-UPR/TMPTA30-BAM and C_f_-UPR/TMPTA30-FLAX, where the moduli drop, appear to be broader, possibly due to the polymer matrix–fiber interactions in the composites. Contrary to the phenomenon in the case of thermal stability of C_f_-UPR/STY and fabricated composites, no significant variations in the *T*_g_ of the C_f_-UPR/TMPTA30 and composites C_f_-UPR/TMPTA30-BAM and C_f_-UPR/TMPTA30-FLAX were observed, as shown in Figure 8c and Table 5. However, a slight increase in the *T*_g_ value was observed with the introduction of fiber reinforcements in the C_f_-UPR/TMPTA30 matrix.

Cole–Cole analysis, a highly preferred model for analyzing the relation between the storage and loss modulus, was carried out to understand the phase behavior of composite samples and relaxation processes within the polymer [88,89,90]. Relaxation occurs because of polymer macromolecule rearrangement, either to adopt a lower energy conformation or to fill free volume. Various factors, such as molecular weight distribution (heterogeneities), free volume distribution, concentration of filler, and degree of crystallinity, directly affect the shape of the Cole–Cole diagram and could be affected by [91]. In the case of the single-relaxation transitions, the diagram appears as a semicircle that intersects the abscissa at the maximum and minimum storage moduli [61]. Otherwise, in the case of material heterogeneity, especially in the hybrid polymer composites, the Cole–Cole analysis results in an irregular shape [60,83]. Figure 8d shows the comparison of Cole–Cole analysis for neat cured C_f_-UPR/STY and C_f_-UPR/TMPTA30, as well as the fabricated composites. The obtained diagrams show imperfect semi-circles, characteristic of the heterogeneous system due to the presence of fibers and interface [92], and are similar for all tested material samples.

### 3.5. Microstructure of the Composite Materials

**Figure 10:**
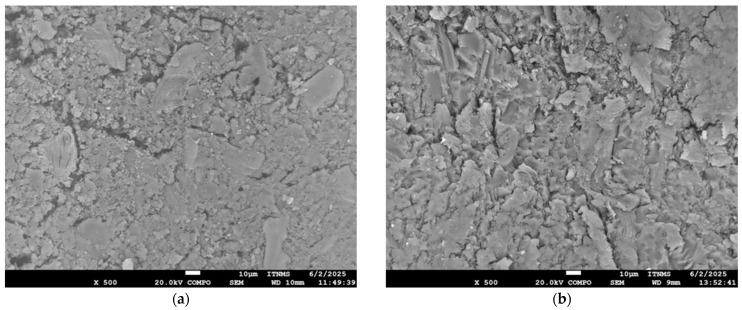

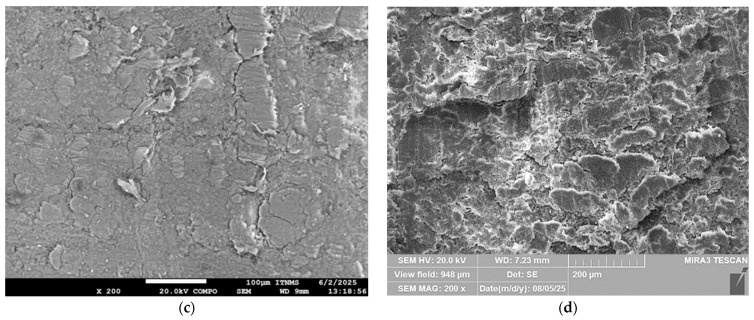
SEM micrographs of the cross-sectional surface of the C_f_-UPR/STY and C_f_-UPR/TMPTA30-based composites: (**a**) C_f_-UPR/STY-BAM, (**b**) C_f_-UPR/TMPTA30-BAM, (**c**) C_f_-UPR/STY-FLAX, and (**d**) C_f_-UPR/TMPTA30-FLAX.

The SEM observation was performed to investigate the tensile fracture surfaces derived from the C_f_-UPR/STY, C_f_-UPR/TMPTA30, and the obtained composites tensile testing, as well as cross-section surfaces. The micrographs obtained are illustrated in Figure 9 (fraction surface) and Figure 10 (cross-section surface).

Figure 9a,b is characterized by a smooth fracture surface of C_f_-UPR/STY and C_f_-UPR/TMPTA30, respectively, with little roughness, which is a characteristic feature of brittle fractures. C_f_-UPR/TMPTA30 presented a mild “ripple” texture, which could indicate a ductile fracture, confirming the strengthening effect of the mechanical properties described in Section 3.4 as a result of the cross-linking network of C_f_-UPR/TMPTA30 through the cross-linking reaction [93].

SEM micrographs Figure 9c–f clearly indicate some detailed information about bamboo and flex fiber orientation and interfacial adhesion between the fibers and polymer matrix. As shown in Figure 9c,e, a large number of pullout fibers, as well as some hollows on the matrix areas, are distinctly observed, which is also indicative in Figure 10a,c as weaker fiber/polymer matrix adhesion. Cracks were detected in the matrix surface of C_f_-UPR/STY-BAM and C_f_-UPR/STY-FLAX (Figure 9c,e), but the cracks were small in size (initiation crack stage). The reason could be the gap between fibers or moderate adhesion between the bamboo/flax fibers and the C_f_-UPR/STY matrix, while in the case of C_f_-UPR/TMPTA30-based composites, cracks are not present, and the polymer matrix has brittle fracture. Present cut-off flax fibers in Figure 9f, meaning fiber breakage when fiber stress level exceeds the local fiber strength, unlike pull-off flex fibers in Figure 9e, indicate good interfacial adhesion between the fibers and C_f_-UPR/TMPTA30, as also confirmed on cross-sectional surfaces in Figure 10b,d. As it can be seen in the SEM micrographs (Figure 9d,f), C_f_-UPR/TMPTA30 causes a significantly better wetting of the hydrophilic and polar natural fibers, especially in the case of flax fibers (Figure 9f), than with the case of C_f_-UPR/STY-based composites (Figure 9c,e), which leads to a significant increase in tensile and flexural strength of C_f_-UPR/TMPTA30-FLAX (Figure 6). Further, C_f_-UPR/TMPTA30-based composites did not show more significant matrix deformation on the tensile fracture surfaces. All these findings are consistent with the results obtained from the mechanical characterization of fabricated C_f_-UPR/STY- and C_f_-UPR/TMPTA30-based composites.

### 3.6. Thermal Analysis

**Figure 11:**
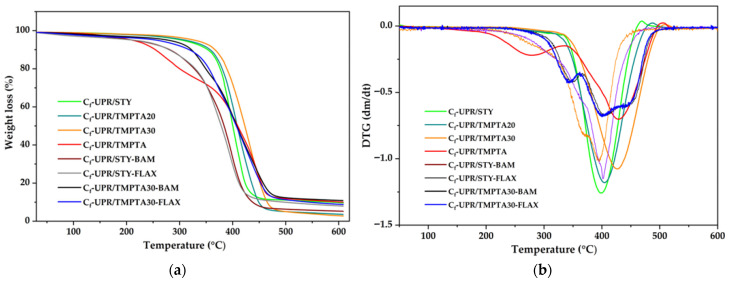
(**a**) Thermogravimetric curves and (**b**) first derivatives for neat cured resins and fabricated constituents.

Analysis of the TGA and DTG curves (Figure 11) of the investigated samples indicates that the degradation mechanism strongly depends on the components present in the formulation. In general, for samples without fiber reinforcement, thermal degradation occurs in two main stages. The first, discrete stage is observed in the temperature range of 25–330 °C, while the second, dominant stage extends from 250 to 550 °C. The mass loss in the first stage is primarily associated with the evaporation of moisture, residual unreacted monomers, and the onset of glycerol degradation [94]. The second stage corresponds to the pyrolytic cleavage of ester bonds within the polymer matrix [95].

For samples with TMPTA, the second degradation stage begins at lower temperatures, around 250 °C, whereas for those with STY it starts at approximately 330 °C. This trend becomes more pronounced with increasing TMPTA content in the resin formulation and can be attributed to the volatilization of unreacted TMPTA. The effect is most clearly observed in the thermogram of the C_f_-UPR/TMPTA sample, where the evaporation of TMPTA results in an additional degradation step in the range of 180–300 °C.

In contrast, the samples reinforced with fibers, both non-woven bamboo fabric (nwBf) and non-woven flax fabric (nwFf), exhibit a somewhat different thermal behavior. The first stage of thermal degradation for these composites begins at around 220 °C. This behavior is likely associated with the degradation of hemicellulose in the fibers, which typically initiates near 200 °C [96].

Further analysis reveals that the residual mass (char) is higher in fiber-reinforced composites compared to neat resin systems, a result attributed to the presence of lignin, which decomposes slowly over a wide temperature range (approximately 250–600 °C) and yields a thermally stable char residue [97]. The formation of this carbon-rich residue reflects greater resistance to decomposition at elevated temperatures. Accordingly, the increased residual mass in lignin-containing composites points to enhanced thermal resilience.

## 4. Conclusions

In this work, a bio-based unsaturated polyester resin was successfully synthesized from camphoric acid, and partial substitution of styrene with trimethylolpropane triacrylate was achieved up to 30%, which is considerably higher than the replacement levels reported in the existing literature. The feasibility of using this resin system for sustainable composite development was further demonstrated through the fabrication of composites reinforced with non-woven bamboo and flax mats. Importantly, the reinforcement materials originated from secondary sources: post-consumer bamboo textiles and flax fibers were obtained as by-products of the textile industry, thereby contributing to resource efficiency and circular material use.

The synthesized resins and obtained composites were comprehensively characterized through rheological, tensile and flexural testing, dynamic-mechanical analysis across a broad temperature range, fracture surface and cross-sectional microscopy, as well as thermal analyses. The results confirmed that the incorporation of TMPTA into the polyester network enhances mechanical performance while maintaining satisfactory thermal stability. Furthermore, the nwFf-reinforced composites exhibited superior tensile and flexural properties compared to both neat resins and bamboo-reinforced systems, underscoring the importance of reinforcement morphology and processing. Generally, the research results showed the following:

- The rheological behavior of the synthesized resins confirmed their Newtonian character, indicating the absence of significant chain entanglements. The observed increase in viscosity with higher TMPTA content can be attributed to the stronger intermolecular interactions arising from the higher polarity of TMPTA compared to styrene, which reduces the diluent’s ability to separate polyester chains.
- The increased water uptake in nwBf and nwFf composites is primarily governed by the hydrophilic character and porous structure of cellulose-based reinforcements. However, reduced water absorption in C_f_-UPR/TMPTA30-based composites indicates that improved interfacial bonding and higher cross-linking density can effectively mitigate moisture sensitivity, enhancing their suitability for applications in humid environments.
- Tensile and flexural testing demonstrated that the incorporation of TMPTA into the polyester matrix enhanced both strength and stiffness due to increased cross-link density, while reinforcement efficiency strongly depended on fiber type and architecture. Non-woven bamboo mats limited load transfer despite good adhesion, whereas flax reinforcements, particularly in combination with C_f_-UPR/TMPTA30, provided superior tensile performance through more effective stress transfer and fiber fracture mechanisms.
- Dynamic-mechanical analysis revealed that flax-reinforced composites exhibited higher storage modulus across both glassy and rubbery regions, indicating restricted chain mobility and efficient stress transfer at the fiber-polymer matrix interface. In contrast, bamboo-reinforced composites showed lower initial stiffness, likely due to fabric architecture and porosity, despite adequate interfacial adhesion. The reduction and broadening of damping factor peaks further confirmed improved interfacial bonding and constrained chain dynamics, particularly in C_f_-UPR/TMPTA30–FLAX composite, which exhibited the highest reinforcement efficiency.
- SEM analysis revealed that C_f_-UPR/STY and its composites exhibited brittle fracture with fiber pullout and voids, indicating weaker fiber–matrix adhesion. In contrast, C_f_-UPR/TMPTA30–FLAX composites showed fiber breakage and ripple textures, confirming stronger interfacial bonding, more efficient stress transfer, and superior reinforcement consistent with mechanical performance results.
- TG analysis showed that neat resins degraded in two main stages, with TMPTA-modified systems exhibiting earlier onset of the second stage due to volatilization of residual TMPTA, while fiber-reinforced composites displayed additional degradation related to hemicellulose at ~220 °C. The higher char yield in nwBf- and nwFf-reinforced composites was attributed to lignin decomposition, indicating improved thermal stability compared to neat C_f_-UPR/STY and C_f_-UPR/TMPTA30.

Overall, the study highlights the potential of camphoric acid-based unsaturated polyester resins, modified with TMPTA, as viable matrices for bio-based composites. When combined with recycled or waste-derived natural fibers, these systems offer a sustainable pathway toward reducing reliance on petroleum-derived feedstocks while maintaining competitive mechanical and thermal performance.

## Figures and Tables

**Table 1:** Water absorption capacity (%) versus time of cured C_f_-UPR’s and prepared polymer composites.

Time (h)	C_f_-UPR/STY	C_f_-UPR/ TMPTA-30	C_f_-UPR/ STY-BAM	C_f_-UPR/ STY-FLAX	C_f_-UPR/ TMPTA30-BAM	C_f_-UPR/ TMPTA30-FLAX
4	0.13	0.12	2.74	2.47	2.22	2.17
8	0.17	0.19	3.02	2.86	2.66	2.59
12	0.20	0.22	3.19	3.01	2.84	2.78
16	0.20	0.23	3.24	3.06	2.9	2.84
20	0.21	0.23	3.3	3.08	2.93	2.88
24	0.21	0.23	3.35	3.11	2.96	2.90

**Table 2:** Comparison of mechanical properties of composites based on UPR matrix reinforced with bamboo or flax non-woven fibers.

Polymer Matrix	Reinforcement	Tensile Strength (MPa)	Young’s Modulus (GPa)	Flexural Strength (MPa)	Flexural Modulus (GPa)	Ref.
UPR with bio-based reactive diluents CINN ^a^, LIM ^b^, MMA ^c^, STY ^d^	-	-	-	21.4 ± 5.4 ^a^ 17.2 ± 2.3 ^b^ 18.5 ± 4.8 ^c^ 29.3 ± 4.2 ^d^	3.26 ± 0.3 ^a^ 2.99 ± 0.4 ^b^ 3.29 ± 0.3 ^c^ 3.99 ± 0.4 ^d^	[8]
UPR (AROPOL 1472/25P Infusion)	Bamboo fibers (treated with 6% NaOH)	21.0	-	44.2	4.0	[51]
UPR (Yukalac 157 BQTN-EX)	-	9.88	-	19.4	-	[52]
	Bamboo fiber *Gigantochloa atter*, 50 mesh, 30% (treated with 6% NaOH)	21.4	-	32.6	-	
UPR (STRUKTOL^®^ POLYVERTEC^®^ 3831) with 2-hydroxyethyl methacrylate (HEMA) diluent	Non-woven flax mat (EcoTechnilin FibriMat ^TM^ F300)	37.9 ± 2.9	4.03 ± 0.3	51.16 ± 7.4	2.95 ± 0.6	[16]
UPR	Bambusa vulgaris, 60% volume fraction, with random orientation (treated with 5% NaOH solution)	16.7	-	-	-	[53]
UPR (bio-based, from camphoric acid) and STY reactive diluent (40 wt.%)	-	18.7 ± 0.7	0.74 ± 0.02	39.5 ± 2.0	3.03 ± 0.03	This work
	Bamboo mat, 30 wt.% (recycled from domestic use)	13.6 ± 0.8	1.33 ± 0.04	26.3 ± 1.1	2.46 ± 0.09	
	Flax mat, 30 wt.% (textile waste flex fibers)	39.8 ± 3.0	2.65 ± 0.08	71.2 ± 5.2	5.99 ± 0.12	
UPR (bio-based, from camphoric acid) and STY + TMPTA reactive diluents (10 + 30 wt.%)	-	25.2 ± 0.7	0.96 ± 0.02	56.1 ± 2.1	3.34 ± 0.03	
	Bamboo mat, 30 wt.% (recycled from domestic use)	13.6 ± 0.7	1.19 ± 0.03	31.4 ± 1.2	2.70 ± 0.06	
	Flax mat, 30 wt.% (textile waste flex fibers)	42.7 ± 2.9	2.76 ± 0.07	95.5 ± 5.9	9.04 ± 0.20	

^a^ Cinnamate (CINN); ^b^ limonene (LIM); ^c^ methyl methacrylate (MMA); and ^d^ styrene (STY).

**Table 3:** Characteristic parameters of the storage modulus in glassy, transition, and rubbery regions, and cross-linking density values for neat cured C_f_-UPR/STY, C_f_-UPR/TMPTA30, and their corresponding composites.

Material	Storage Modulus, *G*′, (MPa) at Certain *T*			*T*_g(*G*′)_, (°C)	υ × 10^−3^, (mol cm^−3^)
	40 °C	100 °C	160 °C		
C_f_-UPR/STY	1137	280	13.4	93.6	3.53
C_f_-UPR/STY-BAM	474	74	24.6	84.6	6.65
C_f_-UPR/STY-FLAX	2006	776	178	93.1	49.8
C_f_-UPR/TMPTA30	1408	211	56.4	51.4	17.6
C_f_-UPR/TMPTA30-BAM	1294	352	132	50.8	40.0
C_f_-UPR/TMPTA30-FLAX	1811	802	484	47.1	139

**Table 4:** Coefficient of reinforcement and volume fraction of constrained region for the obtained composites.

Material	Coefficient of Reinforcement, *C*	Volume Fraction of Constrained Region, *C*_υ_ (%)	Volume Fraction of the Fibers, *V*_f_
C_f_-UPR/STY	-	1.18	-
C_f_-UPR/STY-BAM	0.227	32.0	0.663
C_f_-UPR/STY-FLAX	0.133	29.9	0.542
C_f_-UPR/TMPTA30	-	4.01	-
C_f_-UPR/TMPTA30-BAM	0.393	26.2	0.678
C_f_-UPR/TMPTA30-FLAX	0.382	55.5	0.548

**Table 5:** Glass transition temperature, damping factor peak height, and adhesion factor of cured neat polyester matrix and obtained composites.

Material	*T*_g(*tan* δ)_, (°C)	*tan* δ Height	Adhesion Factor, *A*
C_f_-UPR/STY	121.1	0.41	-
C_f_-UPR/STY-BAM	104.9	0.25	0.809
C_f_-UPR/STY-FLAX	115.5	0.26	0.386
C_f_-UPR/TMPTA30	100.9	0.19	-
C_f_-UPR/TMPTA30-BAM	102.4	0.14	1.288
C_f_-UPR/TMPTA30-FLAX	105.0	0.08	−0.069

## Data Availability

The original contributions presented in this study are included in the article/Supplementary Materials. Further inquiries can be directed to the corresponding author.

## Supplementary Materials

The following supporting information can be downloaded at: https://www.mdpi.com/article/10.3390/polym17223038/s1, Scheme S1: Synthesis of Camphoric acid (C_f_A) from Camphor; Figure S1: (a) Storage modulus, (b) loss modulus and (c) damping factor characteristics for C_f_-UPR/STY and C_f_-UPR/TMPTA30 before and after post-curing at 100 °C and 110 °C, respectively, and (d) DMA parameters for neat cured C_f_-UPR/TMPTA; Figure S2: ^1^H and ^13^C NMR spectra of TMPTA; Figure S3: ^1^H and ^13^C NMR spectra of camphoric acid; Figure S4: 2D and 3D stereoisomers of camphoric acid; Figure S5: 2D structure of the most convenient structure of synthesized resin with atom numbering; Figure S6: Water absorption of synthesized C_f_-UPRs and fabricated composites in by wt.%; Figure S7: Dependence of applied load vs. displacement in three-point bending test; Figure S8: Amplitude sweep curves for determination of strain amplitude value in LVR (a) for C_f_-UPR/STY and correspondent composites, and (b) for C_f_-UPR/TMPTA30 and correspondent composites; Figure S9: Frequency sweep curves for C_f_-UPR/STY, C_f_-UPR/STY-BAM, C_f_-UPR/STY-FLAX, C_f_-UPR/TMPTA30, C_f_-UPR/TMPTA30-BAM and C_f_-UPR/TMPTA30-FLAX; Table S1: Product technical data of flax and bamboo mats; Table S2: Physico-chemical properties of synthesized C_f_-UPRs; Table S3: Cellulose, hemicellulose, and lignin content in non-woven bamboo and flax mats.

## Abbreviations

The following abbreviations are used in this manuscript:

UPR	Unsaturated Polyester Resin
TMPTA	Trimethylolpropane Triacrylate
STY	Styrene
DMA	Dynamic-Mechanical Analysis
MMA	Methyl Methacrylate
C_f_A	Camphoric Acid
nwBf	Non-Woven Bamboo Fabric
nwFf	Non-Woven Flax Fabric
PG	Propylene Glycol
Gly	Glycerol
MA	Maleic Anhydride
AV	Acidic Value
FTIR	Fourier-Transform Infrared Spectroscopy
NMR	Nuclear Magnetic Resonance
SEM	Scanning Electron Microscope
CoAc	Cobalt Octoate
MEKP	Methyl Ethyl Ketone Peroxide
PTFE	Polytetrafluoroethylene
LVR	Linear Viscoelastic Region
G′	Storage Modulus
G″	Loss Modulus
tan δ	Loss Factor (Damping Factor)
T_g_	Glass Transition Temperature
υ	Cross-Linking Density
C	Coefficient of Reinforcement
C_υ_	Volume Fraction of Constrained Region
V_f_	Volume Fraction of the Fibers
A	Adhesion Factor
TGA	Thermogravimetric Analysis
DTG	Derivative Thermogravimetry
