# Supplementary material for: From Nature to Function: Green Composites Using Camphoric Acid-Based Unsaturated Polyester Resin and Bamboo/Flax Non-Woven Reinforcements
Source: Polymers (Basel). 2025 Nov 17;17(22):3038. doi: 10.3390/polym17223038 (PMC12655930; doi:10.3390/polym17223038)
Supplement: Supplementary file 1 [file polymers-17-03038-s001.zip › polymers-3981572-supplementary.pdf]

Supplementary Materials

From Nature to Function: Green Composites Using Camphoric Acid-Based Unsaturated Polyester Resin and Bamboo/Flax Non-Woven Reinforcements

Slavko Mijatov [1,2,*], Sanja Savić [1], Saša Brzić [2], Stefan Ivanović [3], Milena Simić [4], Milena Milošević [5] and Aleksandar Marinković [6]

[1] Department of Organic Chemical Technology, Faculty of Technology and Metallurgy, University of Belgrade, 11120 Belgrade, Serbia; sseslija@tmf.bg.ac.rs
[2] Department of Materials and Protection, Military Technical Institute, 11030 Belgrade, Serbia; sasabrzic@gmail.com
[3] Department of Chemistry, Institute of Chemistry, Technology and Metallurgy—National Institute of the Republic of Serbia, University of Belgrade, 11000 Belgrade, Serbia; stefan.ivanovic@ihtm.bg.ac.rs
[4] Department of Organic Chemistry, Faculty of Pharmacy, University of Belgrade, 11000 Belgrade, Serbia; milena.simic@pharmacy.bg.ac.rs
[5] Department of Ecology and TechnoEconomics, Institute of Chemistry, Technology and Metallurgy—National Institute of the Republic of Serbia, University of Belgrade, 11000 Belgrade, Serbia; milena.milosevic@ihtm.bg.ac.rs
[6] Department of Organic Chemistry, Faculty of Technology and Metallurgy, University of Belgrade, 11120 Belgrade, Serbia; marinko@tmf.bg.ac.rs
* Correspondence: slavko.mijatov@yahoo.com or slavko.mijatov@mod.gov.rs

S1. Materials and Methods

S1.1. Materials

Hydroquinone (HQ), phenylethene (styrene), and dimethylbenzene (xylene: mixture of *-ortho*, *-meta*, and *-para* isomers), and tetrabutyl titanate (TBT), used for synthesis of unsaturated polyester resin, were obtained from Merck. Chemical for composite material preparation, such as methyl ethyl ketone peroxide (MEKP), and cobalt octoate, were supplied from Sigma-Aldrich (Darmstadt, Germany). Camphor was kindly provided by RMD18 Thermoplastic Ltd, Užice, Serbia.

Non-woven flax mat as bio-sourced technical material produced from waste flax fibers, was obtained from EcoTechnilin, France. Further on, non-woven bamboo mat, in the form of bamboo sheets produced for domestic usage, was obtained from Organic Center, Belgrade, Serbia. It represents mat material made from 100% sustainable sources, and in this research it was used as recycled material after primary use. The reuse of these mats not only contributes to environmental protection but also adds value to materials that would otherwise be discarded. Products description properties are shown in Table S1.

Table S1. Product technical data of flax and bamboo mats.

	**Flax**	**Bamboo**
Fiber orientation	Quasi isotropic	Quasi isotropic
Weave	Non-woven	Non-woven
Colour	Brown	White
Composition	100% recycled flax	90% bamboo fibers, 10% PLA
Areal weight (g m^{-2})	100	120
Thickness (mm)	0.20	0.40
Source	Waste flax fibers from textile industry	Waste bamboo sheets from domestic use

S1.2. Preparation of camphoric acid

Natural Camphor can be obtained *via* the distillation of the wood of camphor laurel tree (*Cinnamomum camphora*) found in Borneo, Taiwan, and East Africa, but also in North Africa. The oxidation of Camphor with nitric acid produces Camphoric acid (C_fA) [1], as given in Scheme S1.

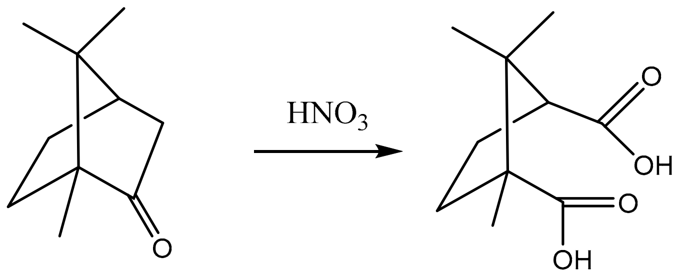

Scheme S1. Synthesis of Camphoric acid (C_fA) from Camphor.

After purification, C_fA is recrystallized from water producing 72% yield of pure product, melting point 184-188 °C. Other raw materials, propylene glycol (PG) and maleic anhydride (MA), used in synthesis of C_f-UPR, are given in our recent publication [2].

S1.3. Synthesis of camphoric based unsaturated polyester resins (C_f-UPRs)

Synthesis of PG, MA, and Glycerol (Gly) from natural resources, along with the selection of molar ratio of chemicals used for bio-based unsaturated polyester resin synthesis was based on procedures published in previous scientific papers [2–8] with a dedicated focus on production of C_f-UPRs with high chain flexibility and adhesivity to natural fibers. Addition of nwBf and nwFf reinforcement would improve mechanical properties and material flexibility. In order to improve contact (interfacial interaction), an appropriate amount of Gly was used in order to introduce hydrophilic side chain functional groups (less reactive secondary hydroxyl). These would be able to create intermolecular hydrogen bonding with hydroxyl groups present at nwBf and nwFf fiber surface.

Thus, the most convenient synthesis of camphoric based unsaturated polyester resins (C_f-UPRs) implies a 1.3:1 of MA:C_fA ratio and it is carried out as follows: in a 500 mL four-necked flask equipped with a reflux condenser, thermometer, nitrogen inlet tube, and Dean-Stark separator, PG (1.25 mol, 98.2 g), Gly (0.25 mol, 22.1 g) and C_fA (0.64 mol, 128 g) were added and heated to 60 °C. Finely crushed MA (0.83 mol, 81.4 g) was gradually added to control the exothermic reaction. At the end of the addition, HQ (0.04 g dissolved in 2.2 g PG) was added, and heating was adjusted at the rate of 10 °C/h to attain 150 °C in the reaction mixture. Separation of reaction was performed by addition of 3 wt.% of the xylene with 0.5 wt.% of the TBT, and heating was continued at the rate of 20 °C/h to reach 210-215 °C and held until almost stoichiometrically generated reaction water was separated (acid value of resin < 12). After, the reaction mixture was cooled to 120 °C, 0.02 g of HQ in 2.2 g of PG was added, and a vacuum was applied to remove low-boiling compounds.

Table S2. Physico-chemical properties of synthesized C_f-UPRs.

Parameter	Value	Unit	Method
Appearance	Transparent yellow	-	-
**C_f-UPR/STY**			
Non-volatile material content	60±1.5	wt. %	ISO 3251
Acid/Hydroxyl value (AV/HV)	4/45	mg KOH/g	ISO 2114
Gel time, 20 °C: 1.5 wt.% MEKP	30-32	min	ISO 2535
Temperature of exotherm peak	82-86	ºC	ISO 2535
Density	1.24±0.28	kg/m^3	ISO 2811
Iodine value	64	-	Wijs
**C_f-UPR/TMPTA30**			
Non-volatile material content	61±1.2	wt. %	ISO 3251
Acid/Hydroxyl value (AV/HV)	5/42	mg KOH/g	ISO 2114
Gel time, 20 °C: 1.5 wt.% MEKP	40-43	min	ISO 2535
Temperature of exotherm peak	<48	°C*	ISO 2535
Density	1.28±0.08	kg/m^3	ISO 2811
Iodine value	62	-	Wijs

Heating at 35 °C

Calculated M_n for C_f-UPR/STY and C_f-UPR/TMPTA30 was 2290 and 2387 g mol^{-1}, respectively. The value of gel time and viscosity of the synthesized UPR resins allow them to be used in practical, *i.e.* commercial, applications.

S1.4. Composite materials production

In order to achieve the desired thermo-mechanical properties of the cross-linked C_f-UPRs, the dynamic-mechanical testing in a wide temperature range was applied as the most reliable method (according to the DMA procedure defined in SM S1.5). C_f-UPR specimens before (C_f-UPR/STY and C_f-UPR/TMPTA30) and after post-curing at 100 °C and 110 °C (C_f-UPR/STY-100 and C_f-UPR/TMPTA30-110, respectively), were subjected to DMA testing in order to define the correct post-curing temperature.

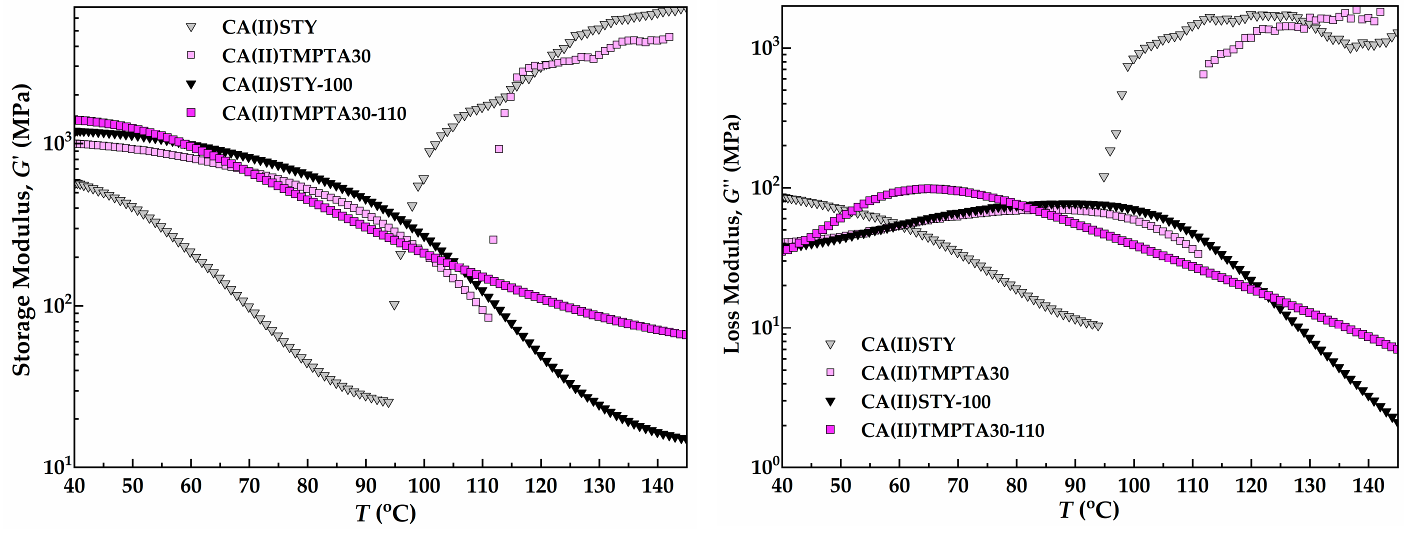

(**a**) (**b**)

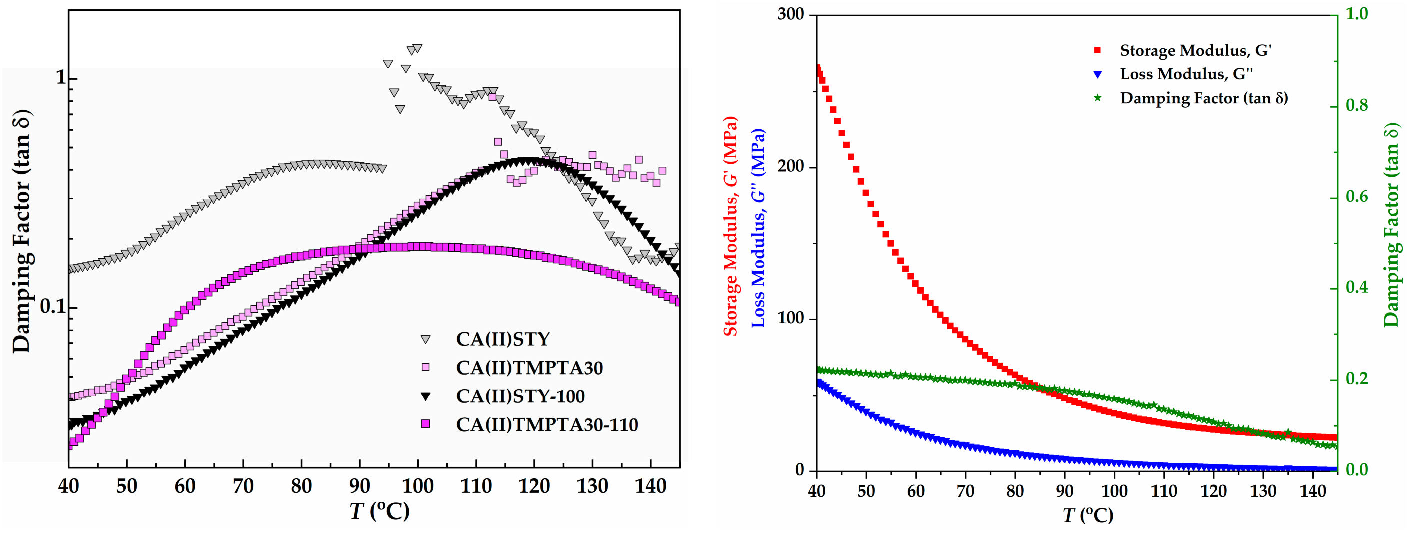

(**c**) (**d**)

Figure S1. (a) Storage modulus, (b) Loss modulus and (c) Damping factor characteristics for C_f-UPR/STY and C_f-UPR/TMPTA30 before and after post-curing at 100 °C and 110 °C, respectively, and (d) DMA parameters for neat cured C_f-UPR/TMPTA.

Storage and loss modulus in Figures S1a and S1b for neat cured C_f-UPR/STY and C_f-UPR/TMPTA30 show irregularities in the temperature range from 40 to 145 °C, which appeared as a sudden jump of modulus values near the glass transition temperature of the materials. It can be attributed to additional cross-linking reactions occurring upon heating. This indicates that the polyester resin was not fully cured prior to testing, leaving functional groups within the network. As the temperature increased, post-curing took place, leading to a denser cross-linked structure. Consequently, the storage and loss modulus remained at a higher, stable level after the transition, confirming the formation of a more rigid network. Thus, it has been found that post-curing at 100 and 110 °C for 1 h is suitable for C_f-UPR/STY and C_f-UPR/TMPTA30, respectively, as well as for obtained nwBf and nwFf reinforced composites.

Figure S1d shows DMA parameters, storage and loss modulus, and damping factor, for neat cured C_f-UPR/TMPTA, with fully replacement of styrene as reactive dulient. The C_f-UPR/TMPTA formulation exhibited nearly constant and comparatively low values of both storage and loss modulus, along with an almost linear and unchanged damping factor (*tan* δ) across the investigated temperature range, and was not selected for further study. Such behavior suggests an absence of significant molecular relaxation or thermal transitions within the analyzed domain, indicating a poorly developed or insufficiently cross-linked network. The lack of variation in viscoelastic response implies that the material does not undergo the characteristic structural relaxations associated with well-defined thermosetting behavior, thus limiting its suitability for composite applications where mechanical stability and thermal performance are critical.

S1.5. Characterization methods

Fourier transforms infrared (FTIR) spectra were recorded with a Nicolet™ iS™ 10 FT-IR Spectrometer (ThermoFisher SCIENTIFIC) equipped with Smart iTR™ Attenuated Total Reflectance (ATR) Sampling accessories, in 400-4000 cm^{-1} range, at 4 cm^{-1} resolution and in 20 scan modes.

^{1}H and ^{13}C NMR measurements were performed on a Bruker Avance III 500 and Bruker Ascend 400 spectrometer equipped with a broad-band direct probe.

The rheological characteristics of the uncured neat unsaturated polyester resins were examined within the linear viscoelastic region, employing a parallel plate (PP25) with a 0.1 mm gap between the plates at 25 °C using Modular Compact Rheometer MCR-302 (Anton Paar GmbH, Austria). The shear rate range was set between 0.1 and 1000 s^{-1} to observe any non-Newtonian behavior. All measurements were in triplicate and the viscosities were averaged and reported. Viscosity measures the liquid flow resistance and is defined as force (*F*) divided by area (*A*); (*F*/*A*). The speed of a moving liquid or (dv/dr) is known as the shear rate, whilst shear stress is the force that acts parallel to the cross-section of the material. The correlation between viscosity (η), shear rate (γ) and shear stress (σ) is expressed in Equation (S1) [9].

$$\eta = \sigma/\gamma \quad \text{(S1)}$$

The following literature methods was applied for cellulose, hemicellulose and lignin determination in the used nwBf and nwFf: Holocellulose [10,11], α-cellulose [11], and klason lignin [12]. Each experiment was repeated three times. Klason lignin is the insoluble part of lignocellulosic materials. The procedure for lignin determination is as follows: the lignocellulosic feedstock was extracted by toluene and ethanol, then 72% concentrated sulfuric acid is added and the reaction is conducted at 30 °C for 4 h; the sulfuric acid is then diluted to 3% and reacted for 2 h with reflux, and the insoluble substrate is weighed as lignin [13,14].

Water absorption behavior of prepared composites was calculated using Equation (S2):

$$\text{Water absorption (\%)} = (W_f - W_i)/W_i \cdot 100 \quad \text{(S2)}$$

Previously, composite samples were oven-dried at 70 °C for 3 h and allowed to cool to the room temperature before testing. The initial dry weight of each sample (W_i) was recorded. The samples were then subsequently soaked in distilled water, and their weights (W_f) were measured at hourly intervals for 4 h, with final weights recorded after 24 h. All measurements were in triplicate and the results were averaged and reported. The composite samples were weighed in analytical scale Mettler Toledo balance accurate to 100 μg.

The tensile strength during the uniaxial stretching of samples was tested according to the standard method ASTM D882 on an INSTRON 1122 testing machine, USA. All tests were performed at 23±2 °C with a stretching rate of 1.0 mm min^{-1}. Five samples of each material were tested and the mean values of tensile strength, deformation, and modulus of elasticity were obtained. The stress-strain curve (σ - ε) is constructed based on the data obtained before the test tube fracture, and the longitudinal modulus of elasticity *E* is obtained based on the straight part of the curve.

Flexural test of cured neat resins and prepared composites was carried out using a three point bending test on the Schenck Trebel RM100 universal testing machine with a load cell of 100 kN. The samples were prepared for the test of dimensions 80 mm × 13 mm × (3±0.2) mm with a recommended span to depth ratio of 16:1 as per ASTM D790. The flexural test was carried out at room temperature with the crosshead speed of 1.5 mm min^{-1}. The radius of punch and supports was 3 mm. The force was applied to the specimen through a fixed loading nose, securing the uniform contact between the loading nose and the specimen across the specimen's width. The tests were conducted until material failure. Flexural strength and flexural modulus were done using Equations (S3) and (S4).

$$\text{Flexural strength} = (3 \cdot F \cdot L)/(2 \cdot b \cdot d^2) \quad \text{(S3)}$$

$$\text{Flexural Modulus} = (m \cdot L^3)/(4 \cdot b \cdot d^3) \quad \text{(S4)}$$

where *F* is ultimate failure load (N), *L* is span length (mm), *b* and *d* are width and thickness of specimen (mm), respectively, and *m* is slope of the tangent to initial line portion of the load-displacement curve. Five specimens of each material were tested and their average values and standard deviation are reported.

The dynamic-mechanical analysis (DMA) of the prepared polymer materials was done by Modular Compact Rheometer MCR-302 (Anton Paar GmbH, Austria) equipped with standard fixtures SRF12 and temperature chamber (CTD–620) which has high temperature stability (±0.1 °C). Torsion mode measurements were performed on rectangular bar specimens with dimensions of (40 x 10 x 3) mm, at temperature range from 40 °C to 180 °C with the heating rate of 2 $°C \cdot min^{-1}$, amplitude of 0.01%, and frequency of 1 Hz. Previously, composite material sample with Ff was tested at a constant temperature (30 °C) and frequency (1 Hz) over a range of strain levels (amplitude strain value ranged up 0.0001% to 0.5%), applying "Amplitude Sweep Test", in order to determine shear stress where material is still in the linear viscoelastic range before reaching the yield stress, so the material is showing reversible viscoelastic behavior. Frequency tests were also carried out on samples of the same geometry at ambient temperature (30.0 ± 0.1 °C), amplitude rate of 0.01% and in frequency range from 0.01 Hz to 50 Hz.

The cross-linking density (υ) of the cured neat C_f-UPR resin and prepared composite materials was calculated from storage modulus, *G*', according to Equation (S5):

$$\upsilon = G'_{Tg+50°C}/R \cdot T \quad \text{(S5)}$$

where υ is the cross-linking density, $G'_{Tg+50°C}$ is the storage modulus (MPa) value at the point where *T* is 50 °C higher then obtained T_g, *R* is the gas constant (8.314 $m^3 \cdot Pa \cdot K^{-1} \cdot mol^{-1}$), and *T* is the temperature in K corresponding to the storage modulus value [2,15].

Characterization of the morphological properties of the fracture surfaces of obtained materials were carried out using a field emission scanning electron microscope FE-SEM MIRA3 Tescan, Oxford, UK, set to 20 kV. Also, using a 30 kV emission scanning electron microscope (FE-SEM, JSM-7001F, Japan), the microstructures of the cross-sectional surfaces of the studied composites were examined (for C_f-UPR/TMPTA30-FLAX). Before imaging, a small layer of Au was sputtered on the samples.

Thermogravimetric analysis (TGA) was conducted using an SDT Q600 TGA/DSC instrument (TA Instruments, New Castle, DE, USA) under a constant nitrogen flow of 30 mL min^{-1}. The temperature program ranged from 25 °C to 600 °C, with a heating rate of 20 °C min^{-1}. Approximately 6.0 ± 0.5 mg of each sample was placed in ceramic crucibles for analysis. To ensure the reproducibility of the results, each sample was analyzed in triplicate.

S2. Results and discussion

S2.1. FTIR and NMR analysis of raw materials, C_f-UPR, and composites

Trimethylolpropane triacrylate (TMPTA) (2,2-bis(prop-2-enoyloxymethyl)butylprop-2-enoate) [16].

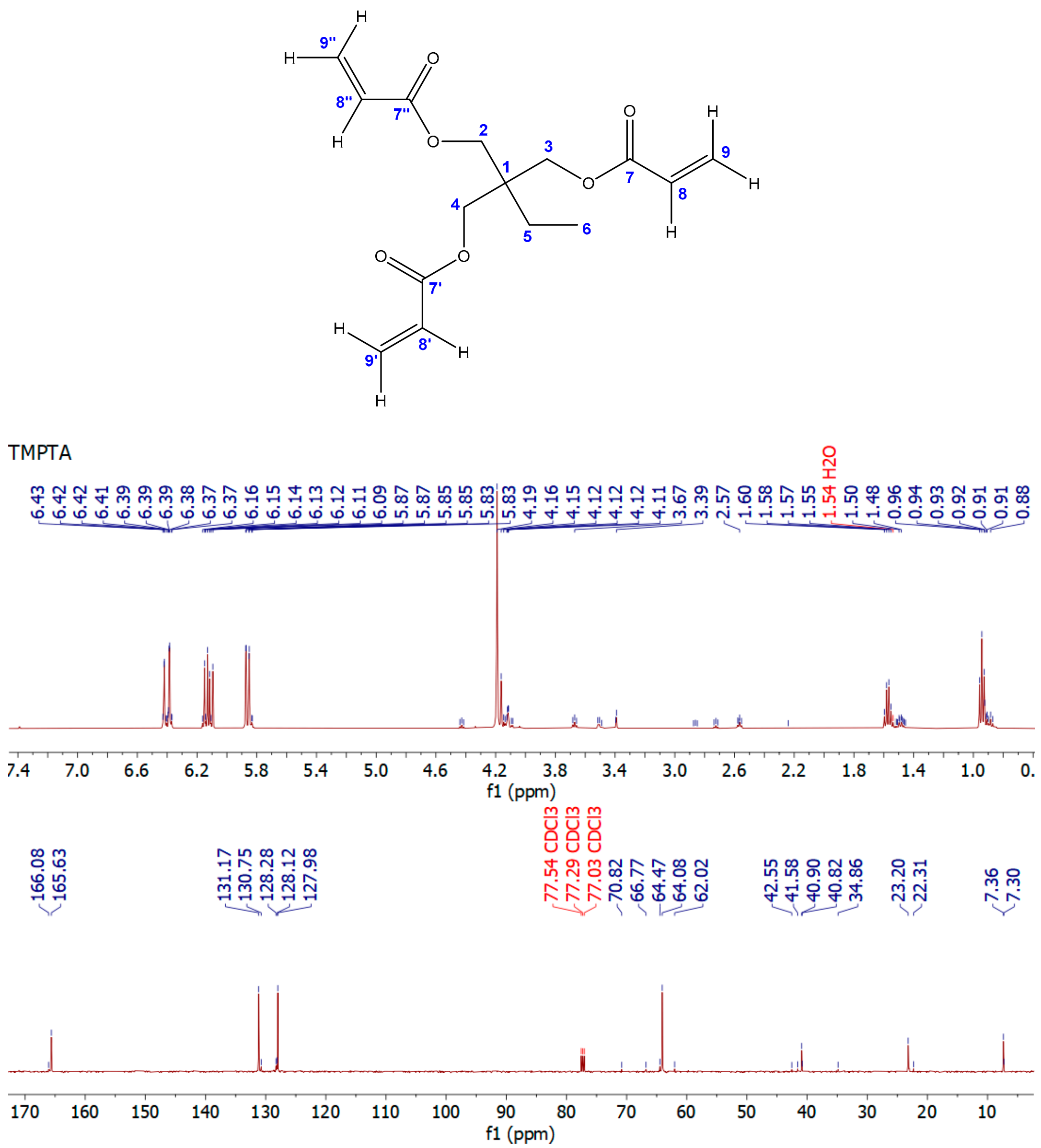

Figure S2. ^{1}H and ^{13}C NMR spectra of TMPTA.

TMPTA peaks assignements: ^{1}H NMR (500 MHz, $CDCl_3$, δ ppm): 0.93-0.96 (*t*, 3H, *J*=8 Hz, H–C6); 1.54-1.60 (*q*, 2H, *J*=8 Hz, H–C5); 4.19 (*s*, 6H, H–C2, H–C3, H–C4); 5.85-5.87 and 6.38-6.42 (*d*, 6H, H–C9, H–C9', H–C9"); 6.09-6.15 (*dd*, 3H, H–C8, H–C8', H–C8"). ^{13}C NMR (126 MHz, $CDCl_3$, δ ppm): 7.35 (C6); 23.20 (C5); 40.90 (C1); 64.08 (C2, C3, C4); 127.98 (C8, C8', C8"); 131.17 (C9, C9', C9"); 165.63 (C7, C7', C7") .

The ^{1}H and ^{13}C NMR spectra of Camphoric acid is given on Figure S3 [17]:

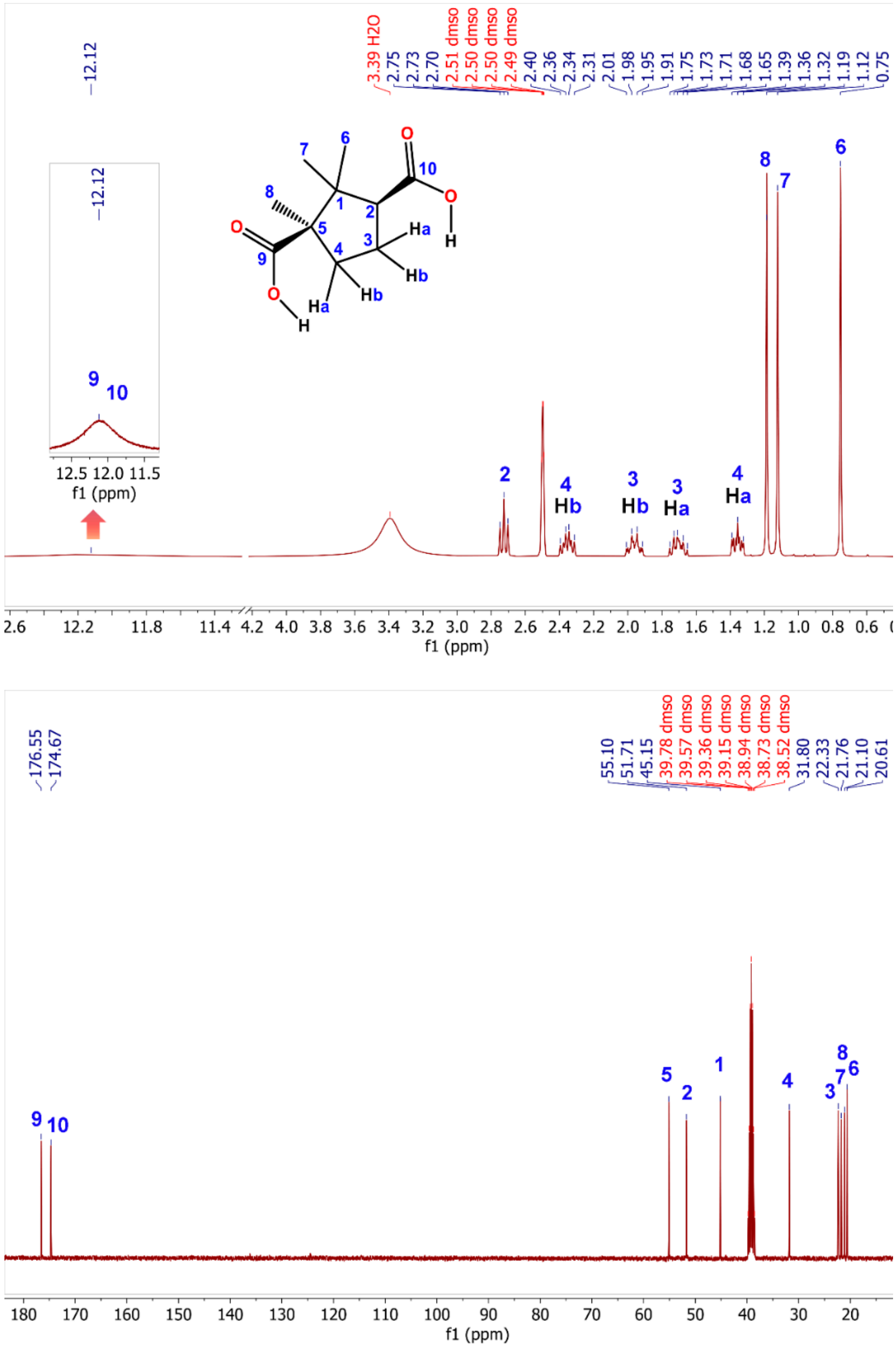

Figure S3. ^{1}H and ^{13}C NMR spectra of camphoric acid.

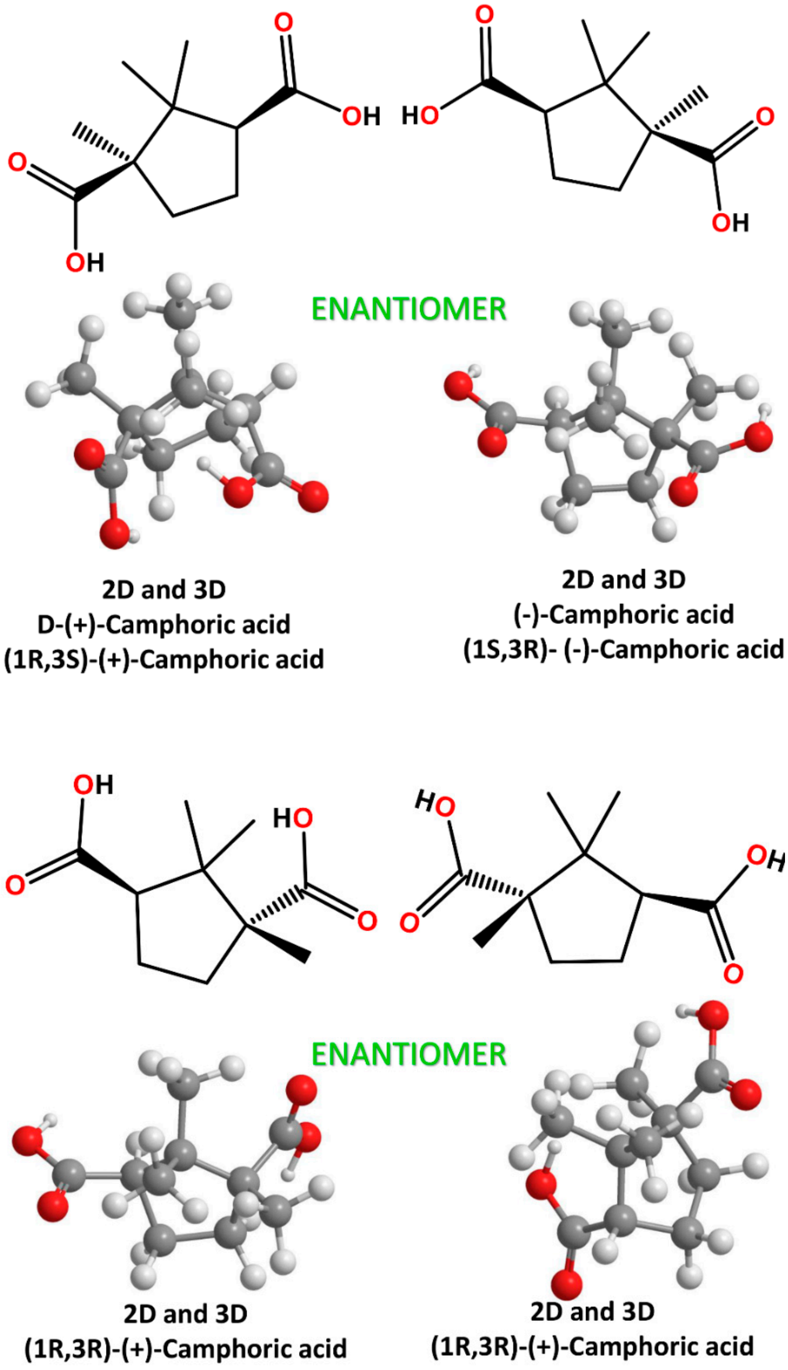

Figure S4. 2D and 3D stereoisomers of camphoric acid.

The results of the ^{1}H and ^{13}C NMR analysis of the camphoric acid with atom numbering (Figure S4) are given in a recent work [17]:

^{1}H-NMR (400 MHz, DMSO-*d6*, δ / ppm): 0,75 (3H, *s*, -C_6H_3), 1,12 (3H, *s*, -C_7H_3), 1,19 (3H, *s*, -C_8H_3), 1,32-1,39 (1H, *m*, -C_4H_a), 1,65-1,75 (1H, *m*, -C_3H_a), 1,91-2,01 (1H, *m*, -C_3H_b), 2,31-2,40 (1H, *m*, -C_4H_b), 2,70-2,75 (1H, *m*, -C_2H), 12,12 (2H, *s*, -C_9-OOH, -C_{10}-OOH); 13C-NMR (100 MHz, DMSO–*d6*, δ / ppm): 20,61, 21,10, and 21,76 (-C_6H_3, -C_7H_3, -C_8H_3), 22,33 (-C_3H_2), 31,80 (-C_4H_2), 45.15 (-C_1), 51.71 (-C_2H), 55.10 (-C_5), 174.67 (-C_{10}OOH), 176.55 (-C_9OOH).

The most plausible structure of synthesized UPR resin is given on Figure S5.

Srtucture I

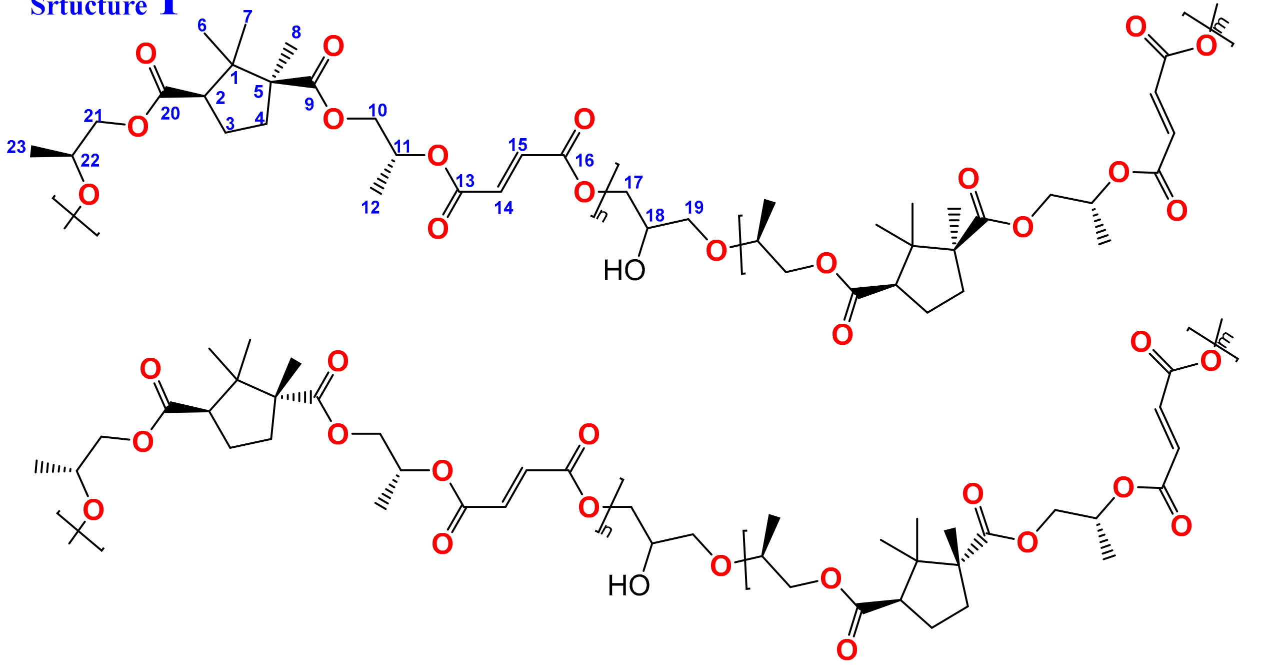

Srtucture II

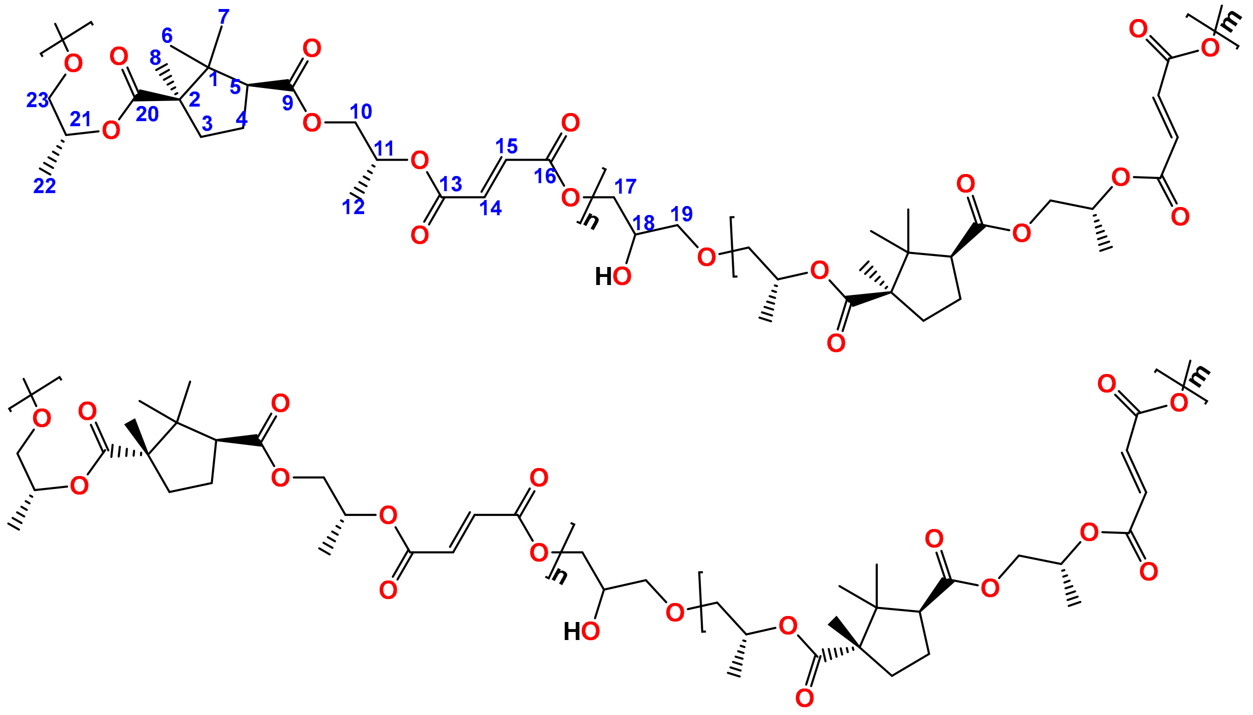

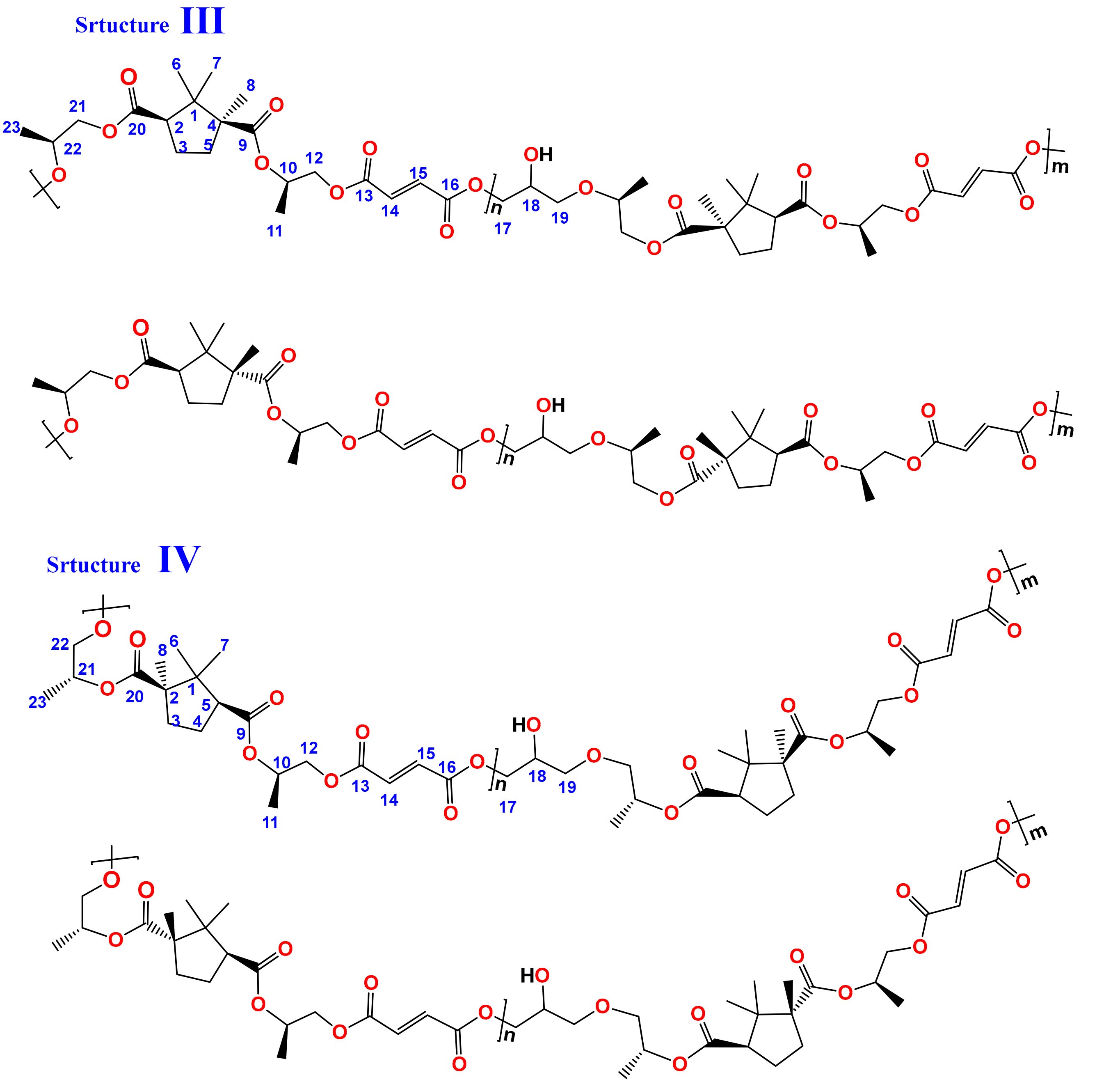

Figure S5. 2D structure of the most convenient structure of synthesized resin with atom numbering.

S2.1.1. Cellulose, hemicellulose and lignin content

According to the applied standard test methods for cellulose, hemicellulose and lignin determination in nwBf and nwFf (from Section S1.5), the results are obtained and presented in Table S3.

Table S3. Cellulose, hemicellulose and lignin content in non-woven bamboo and flax mats.

Component	Bamboo mat, %	Flax mat, %
Cellulose	45	66
Hemicellulose	21	17
Lignin	22	4

S2.2. Water absorption

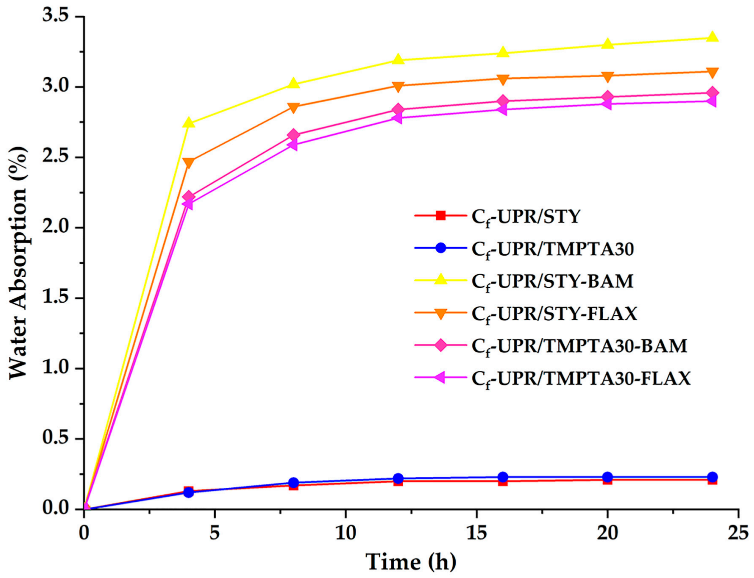

Figure S6. Water absorption of synthesized Cf-UPRs and fabricated composites in by wt.%.

S2.3. Mechanical and dynamic-mechanical properties of cured neat Cf-UPRs and composites

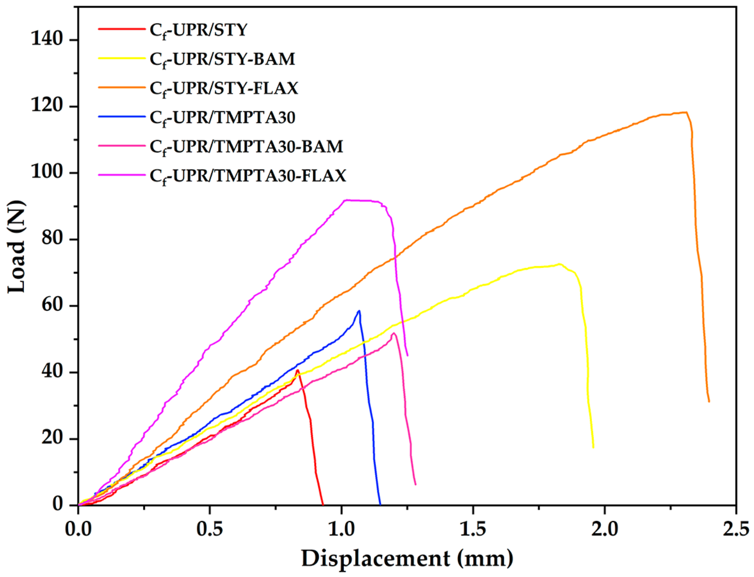

Figure S7. Dependence of applied load *vs*. displacement in three point bending test.

S2.3.1. Dynamic-mechanical properties

After performing DMA test at a constant temperature (30 °C) and frequency (1 Hz) over a range of strain levels (amplitude strain value ranged up 0.0001% to 0.5%), from the results of storage (*G*') and loss (*G*'') modulus shown in Figure S8a for Cf-UPR/STY and correspondent composites, while in Figure S8b for Cf-UPR/TMPTA30 and correspondent composites, it was determined that the strain amplitude for all obtained materials, as the point at which the LVR is exceeded, was 0.01%.

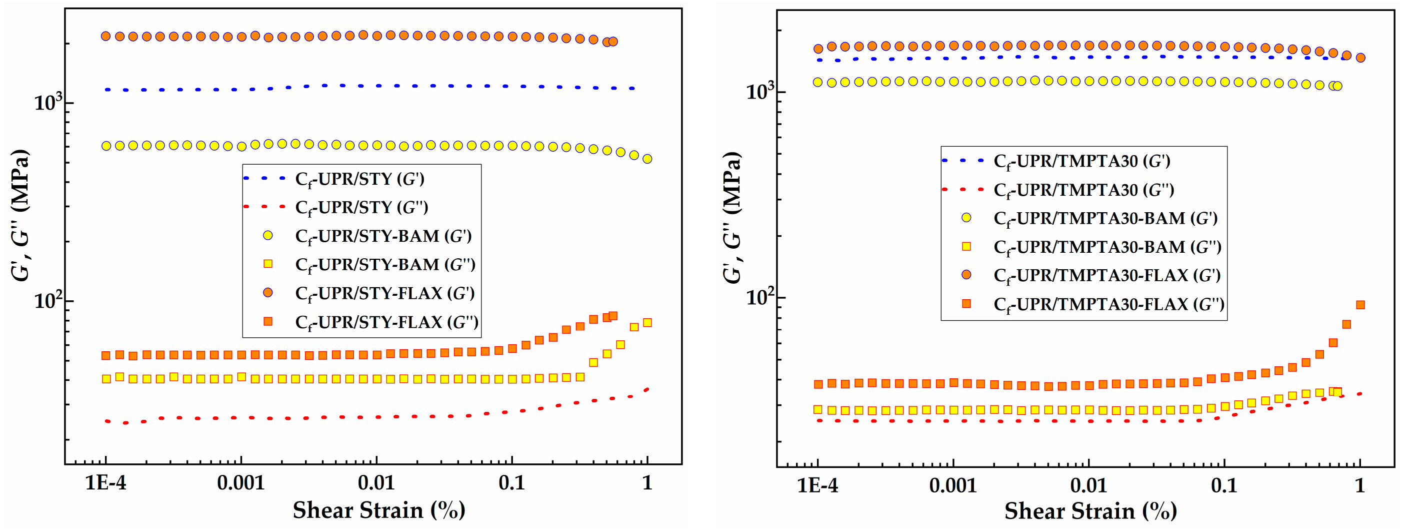

(**a**) (**b**)

Figure S8. Amplitude sweep curves for determination of strain amplitude value in LVR (a) for Cf-UPR/STY and correspondent composites, and (b) for Cf-UPR/TMPTA30 and correspondent composites.

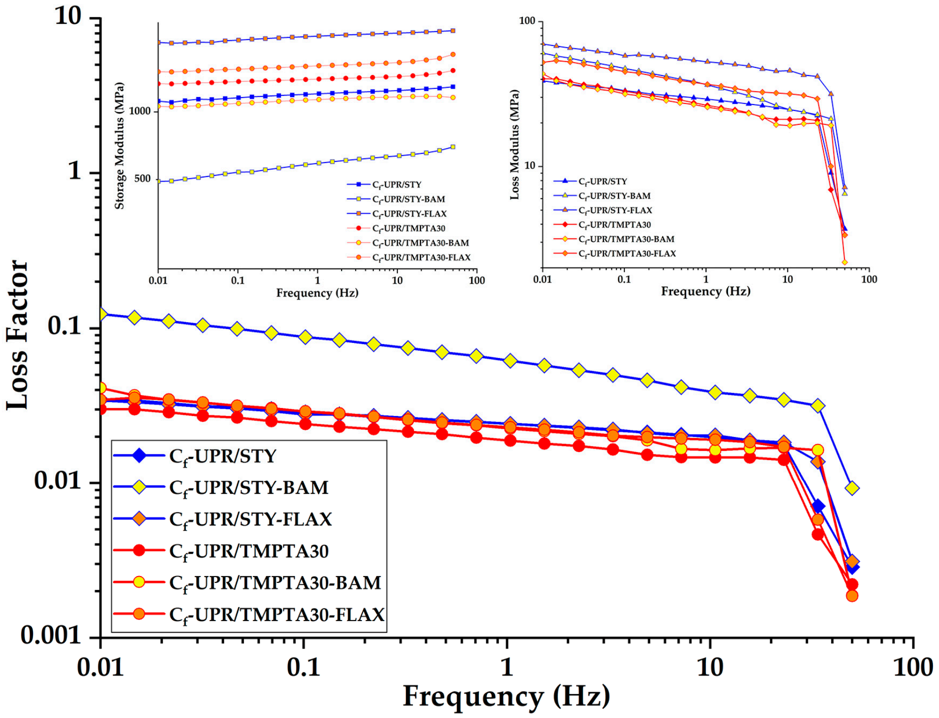

Figure S9. Frequency sweep curves for Cf-UPR/STY, Cf-UPR/STY-BAM, Cf-UPR/STY-FLAX, Cf-UPR/TMPTA30, Cf-UPR/TMPTA30-BAM and Cf-UPR/TMPTA30-FLAX.

The Payne effect test [18], meaning frequency sweep test, was done at 30 °C (Figure S9) and indicates that the synthesized Cf-UPRs, as well as obtained composites, are resilient to viscous deformation and having almost the same loss factor values through frequency sweep, proving the stiff nature of the polymer materials. Except of these characteristics is composite reinforced with nwBf, which can be linked to the structure in the same way as other mechanical features of composites.

References

[1] Nsengiyumva O, Miller SA. Synthesis, characterization, and water-degradation of biorenewable polyesters derived from natural camphoric acid. Green Chem **2019**, 21, 973–8. https://doi.org/10.1039/C8GC03990A.

[2] Embirsh HSA, Stajčić I, Gržetić J, Mladenović IO, Anđelković B, Marinković A, et al. Synthesis, Characterization and Application of Biobased Unsaturated Polyester Resin Reinforced with Unmodified/Modified Biosilica Nanoparticles. Polymers (Basel) **2023**, 15, 3756. https://doi.org/10.3390/polym15183756.

[3] Rusmirovic JD, Trifkovic KT, Bugarski B, Pavlovic VB, Dzunuzovic J, Tomic M, et al. High performances unsaturated polyester based nanocomposites: Effect of vinyl modified nanosilica on mechanical properties. Express Polym Lett **2016**, 10, 139–59. https://doi.org/10.3144/expresspolymlett.2016.14.

[4] Salah Adeen Embirsh H, Vuksanović MM, Mladenović IO, Knežević N, Milošević M, Mijatov S, et al. Unsaturated polyester resin based composites: A case study of lignin valorisation. Chemosphere **2024**, 362, 142144. https://doi.org/10.1016/j.chemosphere.2024.142144.

[5] Kovačević T, Rusmirović J, Tomić N, Marinović-Cincović M, Kamberović Ž, Tomić M, et al. New composites based on waste PET and non-metallic fraction from waste printed circuit boards: Mechanical and thermal properties. Compos Part B Eng **2017**, 127, 1–14. https://doi.org/10.1016/j.compositesb.2017.06.020.

[6] Rusmirović JD, Ivanović JZ, Pavlović VB, Rakić VM, Rančić MP, Djokić V, et al. Novel modified nanocellulose applicable as reinforcement in high-performance nanocomposites. Carbohydr Polym **2017**, 164, 64–74. https://doi.org/10.1016/j.carbpol.2017.01.086.

[7] Rusmirović JD, Kovačević TM, Brzić SJ, Marinković AD. Cross-Linkable Bio and Mineral Fillers for Reactive Polymer Composites: Processing and Characterization. React. Funct. Polym. Vol. Two, Cham: Springer International Publishing; **2020**, p. 135–63. https://doi.org/10.1007/978-3-030-45135-6_6.

[8] Rusmirović JD, Rančić MP, Pavlović VB, Rakić VM, Stevanović S, Djonlagić J, et al. Cross-Linkable Modified Nanocellulose/Polyester Resin-Based Composites: Effect of Unsaturated Fatty Acid Nanocellulose Modification on Material Performances. Macromol Mater Eng **2018**, 303. https://doi.org/10.1002/mame.201700648.

[9] Mudri NH, Abdullah LC, Aung MM, Biak DRA, Tajau R. Structural and Rheological Properties of Nonedible Vegetable Oil-Based Resin. Polymers (Basel) **2021**, 13, 2490. https://doi.org/10.3390/polym13152490.

[10] Mertoglu Elmas G, Yilgor N. Chemical and thermal characterizations of Pinus sylvestris and Pinus pinaster. BioResources **2020**, 15, 3604–20. https://doi.org/10.15376/biores.15.2.3604-3620.

[11] Rowell RM. Handbook of Wood Chemistry and Wood Composites. Second Edi. CRC Press, **2012**.

[12] Standard Specific Interest Group for this Test Method TAPPI. Acid-Insoluble Lignin in Wood and Pulp - T 222 om-02, **2002**.

[13] Sluiter A, Hames B, Ruiz R, Scarlata C, Sluiter J, Templeton D, et al. Determination of Structural Carbohydrates and Lignin in Biomass: Laboratory Analytical Procedure (LAP). Denver West Parkway Golden, Colorado, **2008** (Revised August 2012).

[14] ASTM AS for T and M. Standard Test Method for Methoxyl Groups in Wood and Related Materials (ASTM D-1166–84), **2013**.

[15] Kim TH, Kim M, Lee W, Kim H-G, Lim C-S, Seo B. Synthesis and Characterization of a Polyurethane Phase Separated to Nano Size in an Epoxy Polymer. Coatings **2019**, 9, 319. https://doi.org/10.3390/coatings9050319.

[16] Salih R, Veličković Z, Milošević M, Pavlović VP, Cvijetić I, Sofrenić I V., et al. Lignin based microspheres for effective dyes removal: Design, synthesis and adsorption mechanism supported with theoretical study. J Environ Manage **2023**, 326, 116838. https://doi.org/10.1016/j.jenvman.2022.116838.

[17] Mijatov S, Vuksanović MM, Knežević N, Anđelković B, Cvijetić I, Milošević M, et al. Mechanical Properties of the Bio-Composites: Effect of Kraft Lignin and Flax Fabric to Camphoric Acid Based Unsaturated Polyester Resin's Reinforcement. Polym Compos **2025**. https://doi.org/10.1002/pc.70028.

[18] Nouigues A, Le Gal La Salle E, Bailleul J-L. Thermo-mechanical characterization of unsaturated polyester/glass fiber composites for recycling. Int J Mater Form **2021**, 14, 153–74. https://doi.org/10.1007/s12289-020-01559-8.
